# New High-Affinity Peptide Ligands for Kv1.2 Channel: Selective Blockers and Fluorescent Probes

**DOI:** 10.3390/cells13242096

**Published:** 2024-12-18

**Authors:** Anastasia A. Ignatova, Elena V. Kryukova, Valery N. Novoseletsky, Oleg V. Kazakov, Nikita A. Orlov, Varvara N. Korabeynikova, Maria V. Larina, Arkady F. Fradkov, Sergey A. Yakimov, Mikhail P. Kirpichnikov, Alexey V. Feofanov, Oksana V. Nekrasova

**Affiliations:** 1 Shemyakin-Ovchinnikov Institute of Bioorganic Chemistry, Russian Academy of Sciences, Moscow 117997, Russia; aignatova_83@mail.ru (A.A.I.); evkr@mail.ru (E.V.K.); kazakov.oleg.v@yandex.ru (O.V.K.); n.orlov858@yandex.ru (N.A.O.); var.kora.3@gmail.com (V.N.K.); marya.larina@gmail.com (M.V.L.); arc1567@gmail.com (A.F.F.); sa-yakimov@yandex.ru (S.A.Y.); kirpichnikov@inbox.ru (M.P.K.); onekrasova@ibch.ru (O.V.N.); 2 Department of Biology, Shenzhen MSU-BIT University, Shenzhen 518115, China; novoseletsky@smbu.edu.cn; 3 Faculty of Biology, Lomonosov Moscow State University, Moscow 119234, Russia

**Keywords:** Kv1.2 channel, peptide blocker, Ce peptides, hongotoxin, fluorescent microscopy, patch clamp, dissociation constant, competitive binding

## Abstract

Advanced molecular probes are required to study the functional activity of the Kv1.2 potassium channel in normal and pathological conditions. To address this, a fully active Kv1.2 channel fused with fluorescent protein mKate2 (K-Kv1.2) was engineered that has high plasma membrane presentation due to the S371T substitution, and hongotoxin 1 (HgTx1) fused with eGFP at the C-terminus (HgTx-G) was produced. HgTx-G and HgTx1 N-terminally labeled with Atto488 fluorophore were shown to be fluorescent probes of Kv1.2 in cells with dissociation constants (*K_d_*) of 120 and 80 pM, respectively. K-Kv1.2 and HgTx-G were used as components of an analytical system to study peptide blockers of the channel and helped to find out that Ce1 and Ce4 peptides from *Centruroides elegans* venom possess high affinity (*K_d_* of 10 and 30 pM) and selectivity for Kv1.2. Using molecular docking and molecular modeling techniques, the complexes of Kv1.2 with HgTx1, Ce1, and Ce4 were modeled, and determinants of the high affinity binding were proposed. New fluorescent probes and selective blockers of Kv1.2 can be used to resolve Kv1.2-related challenges in neuroscience and neuropharmacology.

## 1. Introduction

Voltage-gated potassium channels from the Kv1 family regulate propagation of action potential in neurons and maintain membrane potential in non-excitable cells. These channels are involved in diverse physiological processes, such as neuronal excitability and muscle contraction, cytokine and hormone secretion, cell cycle progression, and apoptosis, which, in turn, are related to a great number of diseases and pathological conditions, including cancer, neurological disorders, neuroinflammation, and autoimmunity [1,2,3,4,5].

Kv1.2 α-subunits, which comprise N- and C-terminal cytoplasmic domains and the central six transmembrane helices, assemble into homotetrameric channels or can form heterotetrameric channels with various Kv1 α-subunits.

Most channels of the Kv1 family are notable for their ability to bind peptide toxins from the venoms of poisonous animals. Peptide toxins have been shown to be useful molecular probes for studying the mechanisms of potassium ion conductance and gating in Kv1 channels, as well as the structure-function relationships between different channels [6,7]. Peptide toxins are used effectively to investigate the role of Kv1 channels in cellular functions either in normal physiology or in diseases and are considered as promising pharmacological agents [8,9,10,11].

Most peptide toxins are pore blockers that occlude the ion-conducting pathway of Kv1 channels by interacting with the external surface of the channel pore. Peptide blockers help to identify the pattern of Kv1 channel expression on the plasma membrane of various cells and to evaluate the role of specific Kv1 isoforms, including Kv1.2, in cellular physiology.

For example, dendrotoxins (DTXs) from snake venom are widely used in functional studies of Kv1.2 and other channels. DTXs have different selectivity for Kv1 channels, ranging from α-DTX, which blocks Kv1.1, Kv1.2, and Kv1.6 channels, to more specific DTX-I, which inhibits Kv1.1 and Kv1.2, and further to DTX-K, which is a highly selective blocker of Kv1.1 channels [12]. Using DTX-I and 4-aminopyridine (a non-selective inhibitor of Kv1 channels), it was shown that reduced presence of the Kv1.2 α-subunits in juxta-paranodal regions of peripheral nerves mediates hyperexcitability in type 2 diabetes mellitus [13]. Blocking DTX-sensitive currents at synaptic terminals of cerebellar basket cells, in which Kv1.1 and Kv1.2 channels are present at high density, helped to reveal the major role of these channels in inhibitory of synaptic transmission in the cerebellar cortex [14]. Localization of channels Kv1.2 and Kv1.1 at unmyelinated dendritic segments, nodes of Ranvier, and cell bodies of cochlear afferent nerve fibers was determined by sequential use of 4-aminopyridine, α-DTX, and immunofluorescence labeling [15]. DTX-K was used to demonstrate that the overexpression of the Kv1.1 channel in the demyelinated optic nerve resulted in reduced neuronal excitability [16].

A combination of peptide blockers with different Kv1-channel specificity was found to be useful for studying sub-cellular localization of Kv1 channels or for discriminating between various subtypes of Kv1 channels. Employing α-DTX, DTX-K, tityustoxin Kα, margatoxin (MgTx), hongotoxin 1 (HgTx1), and agitoxin-1, the key role of Kv1.2 and some other Kv1 channels was identified in the regulation of dopamine release at axonal terminals in the striatum [17,18], as well as spiking activity of midbrain dopamine neurons [19]. Both results suggest that Kv1.2 channels can contribute to severe brain disorders, including Parkinson’s disease, that are associated with impaired dopamine release.

Scorpion toxins HgTx1 and MgTx, which have a high affinity for the Kv1.1–Kv1.3 channels, have been used as probes after they were labeled with radioactive isotopes [20,21,22]. Using ^125^I-HgTx1(A19Y/Y37F) in combination with specific anti-Kv1.2 antibodies, it was shown that the Kv1.2 α-subunit was prevalent within heteromeric Kv1 channels of rat brain membranes [20]. Monoiodinated HgTx1 was used as a radioligand probe in the studies of KcsA-Kv1 chimeric channels to characterize their ability to bind different peptide toxins [22].

Following the trend of replacing radioactive probes with fluorescent ones in scientific research, HgTx1-A19C was labeled with sulfhydryl-reactive Cy3-, Cy5-, and Alexa-dyes, and the obtained derivatives were used to visualize Kv1 channels in rat brain sections by fluorescence microscopy [23]. HgTx1 N-terminally labeled with Atto-488 fluorophore (A-HgTx1) was used to characterize membrane localization of Kv1.1 and Kv1.3 channels heterologously expressed in mammalian cells and to determine affinities of some toxins to these channels [24,25]. Tityustoxin labeled with the Atto-594 fluorophore was used to study the regulatory mechanisms of cell-surface expression of Kv1.2 in cells of the rat cerebellum [26].

Definitely, selective blockers of specific isoforms of Kv1 channels may increase the relevance and power of peptide blockers as molecular probes for the channels in various physiological studies. Many peptide blockers with enhanced selectivity for the Kv1.2 channel were found [27], and a few of them possess both high affinity and remarkable selectivity for the Kv1.2 channel. Among such peptides, there are natural peptides from scorpion venoms, including mesomartoxin (α-26.4) [28], MeKTx11-1 (α-1.16) [29], toxin CboK7 [30], and an engineered mutant ChTx(M29I) [31].

Considering the physiological significance of the Kv1.2 channel and potential benefit for the application of specific molecular probes of the Kv1.2 channel in neurobiology, we report on the properties of synthetic A-HgTx1 and engineered by us GFP-tagged HgTx1 (HgTx-G) as fluorescent ligands of Kv1.2 channels in living cells; on the design of the red fluorescent Kv1.2 channel with increased localization in the plasma membrane of mammalian cells; on the development of the analytical system for fluorescent detection and study of peptide blockers of the Kv1.2 channel. Using this system, we characterize recombinant peptides Ce1 and Ce4 originating from the venom of the scorpion *Centruroides elegans* as the high-affinity selective blockers of the Kv1.2 channel. Molecular models of the complexes of HgTx1, Ce1, and Ce4 are presented, and intermolecular interactions that provide high-affinity binding of these peptides to the Kv1.2 channel are discussed.

## 2. Materials and Methods

### 2.1. Reagents

Reagents used are: A-HgTx (Smartox Biotechnology, Saint Egrève, France); transfection reagent GenJector-U (Molecta, Moscow, Russia); LysoTracker Green (LTG) and ER Tracker Green (ERTG) (ThermoFisher Scientific, Waltham, MA, USA); NBD-labeled C6-Ceramide (NCer) and transferrin labeled with the CF488A dye (TR488) (Biotium, Fremont, CA, USA); bovine serum albumin (BSA) and rhodamine 123 (Rh123) (Merck, Darmstadt, Germany). Production and purification of the tobacco etch virus (TEV) protease were performed as described previously [32].

### 2.2. Construction of Expression Plasmids

The *KCNA2* gene of the human Kv1.2 channel (accession number NP_004965) was a gift from Dr. A. Vassilevski. The *KCNA2* gene was amplified by the polymerase chain reaction (PCR) with oligonucleotide primers Kcna2-f1 and Kcna2-r1 (Table 1) and cloned into BglII/HindIII sites of the pmKate2-C expression vector (Evrogen, Moscow, Russia). The obtained plasmid pmKate2-KCNA2 encoded the wild-type Kv1.2 channel tagged N-terminally with mKate2 (K-Kv1.2wt).

The Kv1.2(S371T) mutant was produced by site-directed mutagenesis of the *KCNA2* gene using primers Kcna2m1-f1 and Kcna2m1-r1 (Table 1). In the first round of PCR, two DNA fragments (1140 and 410 bp in length) were obtained separately with two pairs of oligonucleotide primers (Kcna2-f1 and Kcna2m1-r1; Kcna2m1-f1 and Kcna2-r1, respectively) using the pmKate2-KCNA2 plasmid as a template. The DNA fragments were fused and amplified in the second round of PCR using terminal Kcna2-f1 and Kcna2-r1 primers. The obtained mutated gene *KCNA2m1* was cloned into BglII/HindIII sites of the pmKate2-C vector to obtain the plasmid pmKate2-KCNA2m1, which coded for mKate2-Kv1.2(S371T) channel (K-Kv1.2).

The mutant Kv1.2(S371T, N469L) channel was generated by site-directed mutagenesis of the *KCNA2m1* gene in two rounds of PCR as described above using mutagenic primers Kcna2m2-f1 and Kcna2m2-r1 (Table 1). First, the 1430 and 120 bp DNA fragments were synthesized separately from pmKate2-KCNA2m1 plasmid using two pairs of primers (Kcna2-f1 and Kcna2m2-r1; Kcna2m2-f1 and Kcna2-r1, respectively), and then the mutant *KCNA2m1m2* gene was obtained in PCR with the terminal Kcna2-f1 and Kcna2-r1 primers. After cloning the *KCNA2m1m2* gene into the pmKate2-C vector at BglII/HindIII sites, the pmKate2-KCNA2m1m2 plasmid was obtained, which coded for the mKate2-Kv1.2(S371T, N469L) channel (K-Kv1.2m2).

The sequences of wild-type and mutated *KCNA2* genes, as well as the mKate2 gene sequence, were confirmed in the created plasmids by Sanger sequencing of both strands (Evrogen, Russia).

### 2.3. Recombinant Peptide Toxins and HgTx-G

HgTx1, ChTx, MgTx, and AgTx2 peptides were expressed and purified as described previously [33]. Ce1 and Ce4 peptides were obtained as described earlier [34].

Concentrations of peptides were measured using UV spectrophotometry at 214 nm using molar extinction coefficients that were published previously [33,34]. Measurements of peptide concentrations were carried out in the aqueous solution of 20% acetonitrile and 0.1% trifluoroacetic acid.

To produce HgTx1 labeled at C-terminus with eGFP, the previously obtained expression plasmid pET23-MalE-L1-AgTx2-L3-GFP was used [35]. The plasmid coded for the hybrid protein MBP-L1-AgTx2-L3-eGFP, in which L1 and L3 are polypeptide linkers and AgTx2 is flanked N-terminally with CS_TEV_. The DNA fragment between KpnI/BamHI sites of this plasmid, which coded for CS_TEV_-AgTx2, was changed for the DNA fragment I coding for CS_TEV_-HgTx1. The nucleotide sequence of the DNA fragment I was as follows:

$$\begin{array}{l} \text{5′-}\text{GGTACC}\underline{\text{GAAAACCTGTATTTTCAG}\text{ACC}}\text{GTGATCGATGTCAAATGCACCAGCCCGAAACAGTGCCTGCCG}\\ \text{CCATGTAAAGCGCAGTTCGGTATTCGTGCCGGCGCAAAATGCATGAACGGTAAATGCAAATGTTATCCG}\\ \text{CAT}\text{GGATCC}\text{-3′,} \end{array}$$

where the flanking KpnI and BamHI sites are shown in italics, the DNA fragment coding for CS_TEV_ is underlined, and the codon ACC (Thr) is underlined and shown in bold. The residue Thr, which occupies the P’ position of non-canonical TEV cleavage site ENLYFQT, overlaps the N-terminal residue of HgTx1 (TVIDVKCTSPKQCLPPCKAQFGIRAGAKCMNGKCKCYPH).

**Figure 1:**
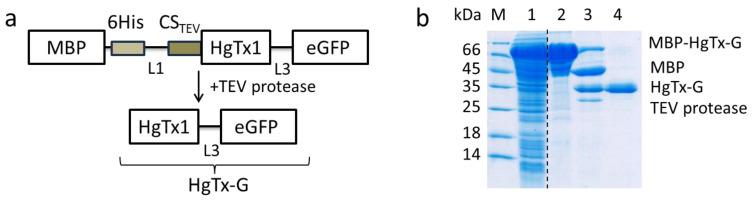
Production of HgTx-G. (**a**) A scheme of MBP-tagged fusion protein produced in *E. coli* cells, which was further cleaved by TEV protease to obtain HgTx-G. CSTEV—TEV protease cleavage site, 6His—six His tag, L1 and L3—polypeptide linkers. (**b**) SDS-PAGE (12%) of the *E. coli* cell lysate with expressed fusion protein MBP-HgTx-G (lane 1), MBP-HgTx-G purified with Ni-affinity chromatography (lane 2), products of MBP-HgTx-G hydrolysis with the TEV protease (lane 3), and purified HgTx-G (lane 4).

The DNA fragment I was PCR-amplified from the plasmid pET23-MalE-L1-HgTx1 [33] using oligonucleotide primers Hg-f1 and Hg-r1 (Table 1) and then cloned into corresponding sites of pET23-MalE-L1-AgTx2-L3-GFP. The obtained plasmid pET23-MalE-L1-HgTx1-L3-GFP coded for the hybrid protein MBP-L1-HgTx1-L3-eGFP (Figure 1a), which was expressed in *E. coli* strain Rosetta-gami(DE3)pLysS and purified from the biomass as described earlier [35]. Hydrolysis of the hybrid protein with TEV protease resulted in the formation of the HgTx1-L3-eGFP ligand (denoted as HgTx-G in the present article), in which the native N-terminal residue of HgTx1 was retained (Figure 1a). To purify the fluorescent ligand after the hydrolysis, the reaction mixture was subjected to Ni-Sepharose chromatography, and the target protein was eluted in the flow-through fractions, whereas the His-tagged proteins, including His6-TEV, were removed [35]. The HgTx-G ligand was desalted on a PD-10 column (GE Healthcare, Chicago, IL, USA) in PBS buffer and then concentrated on an Amicon Ultra 10 kDa filter (Millipore, Merck KGaA, Darmstadt, Germany) to 1.5 g/L. The protein was stored at 4 °C in the presence of 0.02% sodium azide. The concentration of the protein was determined using the molar extinction coefficient for eGFP (56,000 M^−1^ cm^−1^ at 489 nm). The SDS-PAGE was used to analyze the protein purification steps (Figure 1b).

### 2.4. CD Spectrum Measurements and Analysis

CD spectra of Ce1 (86 µM), Ce4 (119 µM), and HgTx1 (36 µM) peptides were measured in a buffer containing 50 mM NaClO_4_, 12.5 mM Na_2_HPO_4_, and 12.5 mM NaH_2_PO_4_ (pH 7.5) using a J-810 spectropolarimeter (Jasco, Tokyo, Japan) and a quartz cell of 100 µm pathlength (Hellma Switzerland AG, Buchs, Switzerland). CD spectra were recorded in the 190–250 nm spectral range (0.2 nm step) and analyzed using the CONTINLL program [36].

### 2.5. Cell Culture and Transfection

Mouse neuroblastoma Neuro2a cells were obtained from the Russian collection of cell cultures (Institute of Cytology RAS, Saint Petersburg, Russia). Cell culturing was carried out as described previously [25]. Cells at a density of 30,000 cells/mL were seeded into 24-well plates (1 mL per well) on circular glass coverslips (10 mm, Gerhard Menzel GmbH, Braunschweig, Germany) precoated with poly-L-lysine (Paneco, Moscow, Russia). Transfection of cells with plasmids was performed using GenJector-U reagent according to the manufacturer’s protocol at 30–40% cell confluence. The experiments were conducted 15–30 h after transfection.

Cellular organelles were stained as described earlier [24,25]. To analyze the binding of Kv1.2 with fluorescent ligands, A-HgTx (0.06–2 nM) or HgTx-G (0.06–0.5 nM) was added to cells in a complete medium for 90–120 min. To analyze competitive binding, HgTx-G (0.5 nM) was added to cells together with the studied peptide (0.1–0.8 nM HgTx1; 0.05–0.8 nM MgTx; 5–20 nM ChTx; 50–400 nM AgTx2; 0.05–0.2 nM Ce1; 0.2–5 nM Ce4) in a complete medium for 90–120 min.

### 2.6. Electrophysiology Measurements

The patch-clamp method (whole-cell configuration) was used to measure membrane ion currents as described earlier [24]. All the measurements were carried out at room temperature in a buffer containing (mM) 140 NaCl, 2.8 KCl, 2 MgCl_2_, 2 CaCl_2_, 10 HEPES, and 10 glucose (pH 7.4). Resistance of the micropipette tip was 6–8 MΩ. The pipette solution contained (mM) 140 KCl, 6 CaCl_2_, 2 MgCl_2_, 2 MgATP, 0.4 NaGTP, 10 HEPES, and 20 BAPTA/KOH (pH 7.3). The holding potential was −40 mV. The membrane potential increased from −70 to +70 mV with the 20 mV step each 20 s and returned to −40 mV. The duration of voltage pulses was 200 ms.

Measurements were carried out in 24–48 h after transfection of cells. HgTx1 (2 nM), HgTx-G (7.5 nM), Ce1 (10 nM), and Ce4 (10 nM) were dissolved in the extracellular buffer.

### 2.7. Confocal Microscopy

Microscopy was carried out using an SP2 confocal microscope with an HCX PL APO 63×/1.2 objective (Leica Microsystems GmbH, Wetzlar, Germany). The size of the confocal diaphragm corresponded to 1 Airy disk. Typical voxel size was 0.06 × 0.06 × 0.9 µm except for serial ligand binding experiments, where its size was 0.23 × 0.23 × 0.9 µm. The fluorescent imaging was performed as follows: excitation at 561 nm, detection at 650–700 nm for the Kv1.2 channels; excitation at 488 nm, detection at 498–535 nm for A-HgTx, HgTx-G, and A-ChTx; excitation at 488 nm, detection at 500–550 nm for probes of cellular organelles. To minimize cross-talk between signals in two-color experiments, sequential scanning was performed.

### 2.8. Quantitative Analysis of Fluorescent Images

Quantitative analysis of the interaction of fluorescent ligands with Kv1.2 was carried out using Image J software (version 1.48, National Institute of Health, Bethesda, MD, USA) as described earlier [24,25]. This analysis was based on calculating the ratio of the fluorescent intensities of the bound ligand to the channel (*R_i_*) in each selected region of interest on the plasma membrane for the confocal images of 20–25 measured cells, followed by calculating the average value (*R_av_*) and standard deviation.

The dissociation constant (*K_d_*) of a fluorescent ligand complex with Kv1.2 was estimated from the dependence of *R_av_* on the concentration *L* of the added fluorescent ligand:

*R_av_*(*L*) = *R_m_L*/(*K_d_* + *L*) (1)

*R_m_* is the maximal *R_av_* value at the saturation of ligand binding.

The peptide concentration (*DC*_50_) displacing 50% of the fluorescent ligand from the complex with Kv1.2 was estimated by the dependence of *R_av_* on the concentration (*C*) of the added peptide at the fixed concentration of the fluorescent ligand:

*R_av_*(*C*)/*R_av_*_0_ = 1/(1 + *C*/*DC*_50_) (2)

*R_av_*_0_ is *R_av_* before peptide addition.

The apparent dissociation constant (*K_ap_*) of the complex between Kv1.2 and the studied peptide was calculated using the Cheng–Prusoff equation:

*K_ap_* = *DC*_50_/(1 + *L*/*K_d_*) (3)

All the experiments were performed in triplicate; data were averaged and presented as mean ± SD.

### 2.9. Molecular Modeling

A structural model of HgTx1 was obtained from the Protein Data Base [37], with the identificator 1HLY [23]. Structural models of Ce1 and Ce4 were predicted by the AlfaFold2 web service [38], identificators AF-P0C161-F1 and AF-P0C164-F1, respectively. Molecular dynamics (MD) simulations (100 ns, water solution) were performed to obtain representative conformations of peptides.

A molecular model of the tetrameric membrane domain of the Kv1.2 channel (amino acid residues 141–430) was obtained with the ColabFold web service [39]. Five models, generated using the ColabFold web service, differed primarily in some unordered regions of the polypeptide chain. The best model ranked #1 according to the ColabFold internal score, was selected for the MD experiment. The channel-lipids system was obtained using the CHARMM-GUI web server [40]. The channel was embedded in a bilayer of ~750 POPC lipids with ~38,000 TIP3P water molecules and 0.15 M of KCl to form a simulation box of ca. 120 × 120 × 120 Å (~178,000 atoms total). One potassium ion was manually placed in the selective filter of the channel to maintain its stability during MD simulation. The system was equilibrated using the standard CHARM-GUI protocol with the gradual removal of constraints. An unrestrained MD simulation of the channel (100 ns) was performed with the CHARMM27 forcefield [41] and Gromacs software (version 2024.3) [42]. The pressure was kept at 1.01325 Bar by the c-rescale method, and the temperature was maintained at 303.15 K by a Berendsen thermostat with a damping coefficient of 1 ps^−1^. Since the symmetry of the channel associated with a fourth-order rotational axis was disturbed during MD simulation, it was artificially restored by averaging the conformations of individual chains. Finally, the representative structure of the channel after the MD and symmetrization procedures was found to be very similar to that in the ColabFold model. Representative conformations of the channel and peptides were used for the molecular docking studies of peptide-channel complexes, which were performed using the ClusPro web server [43]. Docking solutions were inspected manually, and those where occlusion of the ion pore with the side chain of an amino acid residue occurred were chosen for further MD simulation. MD simulation of the complexes (two independent trajectories: 10 ns with peptide position restraining followed by 100 ns unrestrained for every trajectory) was performed to study peptide-channel contacts. Pressure, temperature, and other parameters were as described above. Long-range electrostatic interactions were evaluated with the smooth Particle Mesh Ewald algorithm. For short-range non-bonded interactions, a cutoff of 12 Å with a switching function at 10.0 Å was used. The integration time step was 2 fs.

## 3. Results

### 3.1. Design of Fluorescent Kv1.2 Channels with Increased Localization in Plasma Membrane

**Figure 2:**
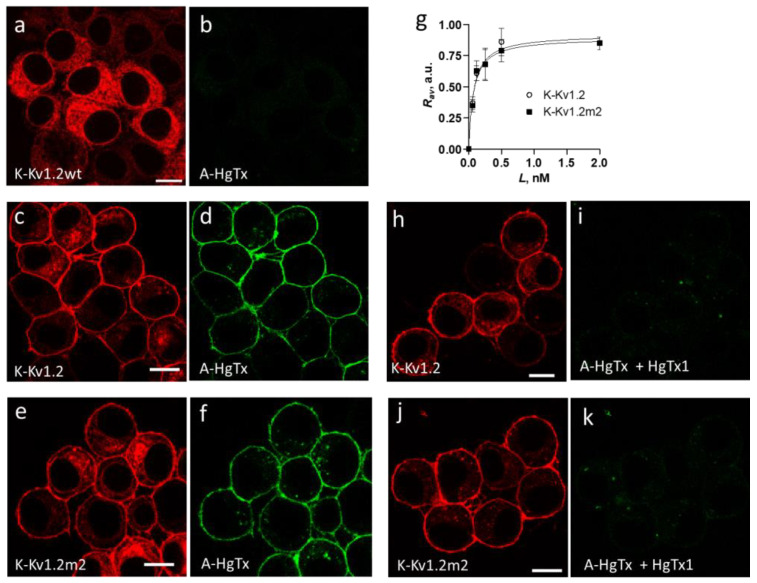
Expression of K-Kv1.2wt, K-Kv1.2, and K-Kv1.2m2 in Neuro2a cells and their interaction with A-HgTx and HgTx1. (**a**–**f**) Binding of A-HgTx to cells that express K-Kv1.2wt (**a**,**b**), K-Kv1.2 (**c**,**d**), or K-Kv1.2m2 (**e**,**f**). Distributions of channels and A-HgTx are shown in red and green, respectively. Cells were incubated with 20 nM (**a**,**b**) or 0.5 nM (**c**–**f**) A-HgTx for 1 h. (**g**) Concentration dependences of A-HgTx binding to K-Kv1.2 and K-Kv1.2m2 that are presented in terms of the dependence of the *R_av_* parameter (see the Section 2.8.) on the concentration *L* of A-HgTx added to cells. Data are averaged over three independent experiments and presented as mean ± SEM (**h**–**k**). Competitive displacement of A-HgTx with HgTx1 from the complexes with K-Kv1.2 (**h**,**i**) and K-Kv1.2m2 (**j**,**k**). Cells were incubated with 0.5 nM A-HgTx and 10 nM HgTx1 for 1 h. Bar is 15 µm.

Neuro2a cells were transiently transfected with the pmKate2-KCNA2 plasmid, which encodes wild-type human Kv1.2 fused at the N-terminus with mKate2 fluorescent protein (K-Kv1.2wt). A considerable part of transfected cells (30–50%) expressed K-Kv1.2wt and demonstrated a web-like pattern of the cytoplasmic distribution of K-Kv1.2wt. This distribution was characterized by the absence of a distinct localization of channels in the plasma membrane (Figure 2a). The addition of a fluorescent ligand of Kv1-channels A-HgTx [24,25] to the cells did not lead to staining of a cell surface, regardless of whether cells expressed K-Kv1.2wt (Figure 2b) or not (Figure S1a,b).

Assuming that the expression of K-Kv1.2wt on the plasma membrane is low, similar to native Kv1.2 [44], a search was conducted for mutations that can improve the presentation of the channel at the membrane. Based on previously published data [44,45], the mutation S371T as well as the combination of the mutations S371T and N469L were considered. Two plasmids were engineered that encoded chimeric proteins K-Kv1.2 and K-Kv1.2m2, in which mKate2 was fused at its C-terminus with Kv1.2(S371T) and Kv1.2(S371T/N469L), respectively [44,45]. The plasmalemma presentation of K-Kv1.2 and K-Kv1.2m2 was increased as compared to K-Kv1.2wt (Figure 2a–f), and A-HgTx stained the plasma membrane of cells that expressed K-Kv1.2 or K-Kv1.2m2 (Figure 2c–f). An excess of pore blocker HgTx1 displaced A-HgTx from the surface of these cells (Figure 2h–k). Definitely, A-HgTx and HgTx1 competed for binding to the same site, and this site was the outer vestibule of the pore of the channels formed by Kv1.2(S371T) or Kv1.2(S371T/N469L) α-subunits. A-HgTx demonstrated similar concentration dependences of binding to K-Kv1.2 and K-Kv1.2m2, with binding saturation at ~0.5 nM A-HgTx (Figure 2g). The dissociation constants (*K_d_*) of the complexes determined using these dependencies were the same for K-Kv1.2 or K-Kv1.2m2 and equal to 80 ± 20 pM.

### 3.2. Intracellular Localization of Kv1.2 Channels

In order to characterize the distribution of K-Kv1.2wt, K-Kv1.2, and K-Kv1.2m2 in the cytoplasm of cells, Neuro2a cells expressing particular fluorescent α-subunits of Kv1.2 channels were stained with fluorescent markers of cellular organelles. The endoplasmic reticulum (ER) was stained with ER Tracker Green (ERTG). Trans-Golgi cisternae were visualized with NBD-labeled C6-ceramide (NCer). Endosomes were stained using transferrin conjugated with the CF 488A fluorophore (TR488). Lysosomes were stained with LysoTracker Green (LTG). Rhodamine 123 (Rh123) was used to visualize mitochondria.

**Figure 3:**
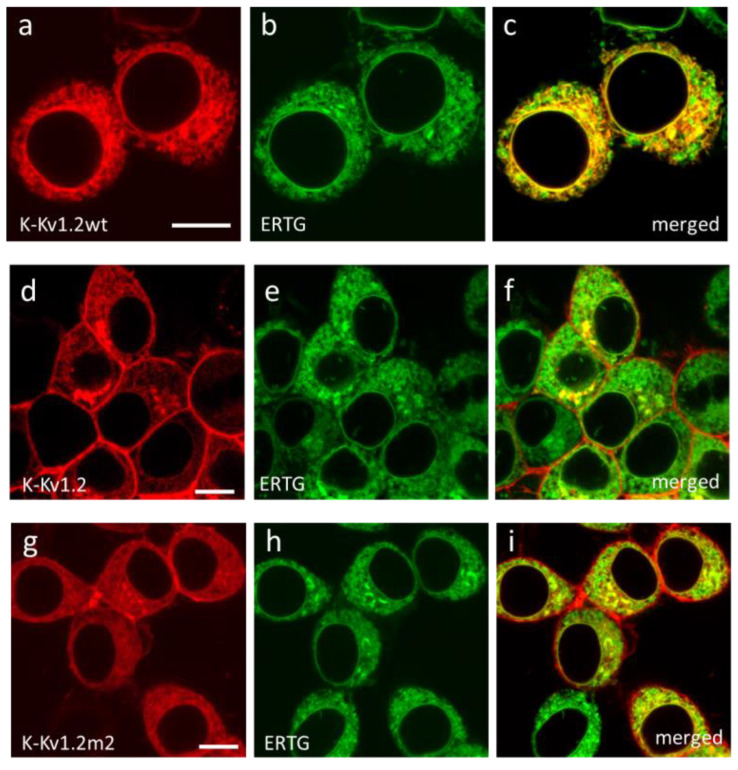
Analysis of localization of K-Kv1.2wt, K-Kv1.2, and K-Kv1.2m2 in the ER of Neuro2a cells. (**a**,**d**,**g**) Confocal images of a distribution of K-Kv1.2wt (**a**), K-Kv1.2 (**d**), and K-Kv1.2m2 (**g**) in cells. (**b**,**e**,**h**) Confocal images of a distribution of the fluorescent ER marker (ERTG) in cells. (**c**,**f**,**i**) Merged images of ERTG and Kv1.2 channels. A yellow color indicates the co-localization of Kv1.2 (red) and ERTG (green) in ER. Bar is 20 µm.

**Figure 4:**
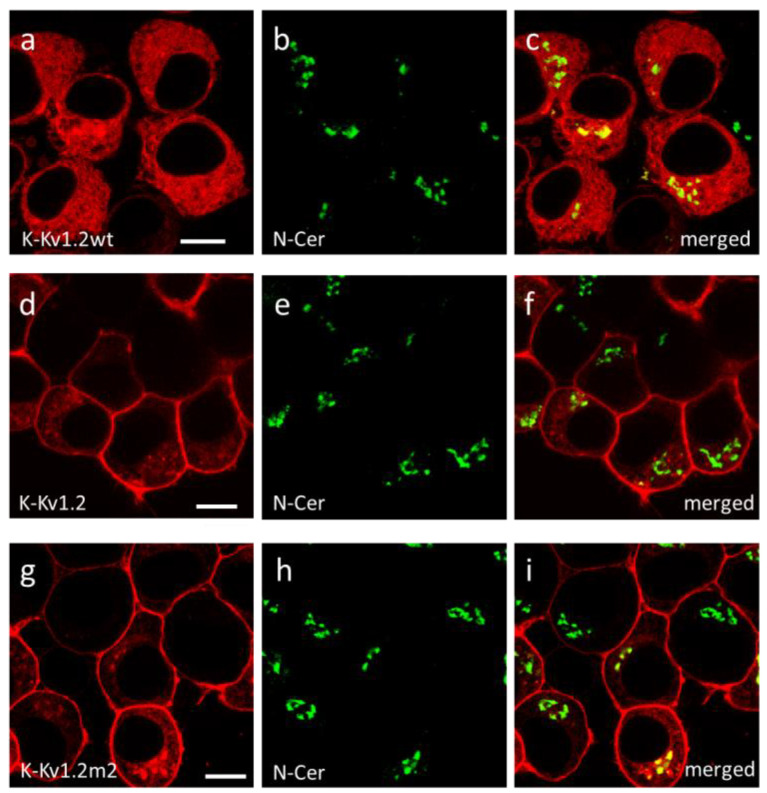
Analysis of localization of K-Kv1.2wt, K-Kv1.2, and K-Kv1.2m2 in the trans-Golgi cisternae of Neuro2a cells. (**a**,**d**,**g**) Confocal images of a distribution of K-Kv1.2wt (**a**), K-Kv1.2 (**d**), and K-Kv1.2m2 (**g**) in cells. (**b**,**e**,**h**) Confocal images of the distribution of fluorescent marker of the trans-Golgi structures (N-Cer) in cells. (**c**,**f**,**i**) Merged images of N-Cer and Kv1.2 channels. A yellow color indicates the co-localization of Kv1.2 (red) and N-Cer (green) in the trans-Golgi network. Bar is 20 µm.

It was found that features of the intracellular distribution of K-Kv1.2wt, K-Kv1.2, and K-Kv1.2m2 subunits are similar. They are predominantly localized in ER (Figure 3) and in the Golgi apparatus (Figure 4), i.e., in two compartments that are involved in the assembly of the channel and its further traffic to the plasma membrane. Considering the accumulation of K-Kv1.2wt in trans-Golgi cisternae and the absence of channels in the plasma membrane, one can suggest that retention of Kv1.2 in the trans-Golgi network is the limiting stage of its membrane traffic. K-Kv1.2 and K-Kv1.2m2 overcome traffic blocking due to a mutation-induced increase in the channel glycosylation in the Golgi apparatus [46,47].

The presence of K-Kv1.2wt, K-Kv1.2, and K-Kv1.2m2 subunits in endosomes and lysosomes is rarely observed (Figures S2 and S3). K-Kv1.2wt, K-Kv1.2, and K-Kv1.2m2 subunits are not able to accumulate in mitochondria (Figure S4).

Since it was found that K-Kv1.2 and K-Kv1.2m2 are similar in terms of expression level, cytoplasmic distribution, plasma membrane presentation, and A-HgTx binding (Figure 2c–f,h,j), further studies were conducted using a protein construct K-Kv1.2 that has the only mutation, S371T.

### 3.3. Electrophysiology Study of K-Kv1.2

**Figure 5:**
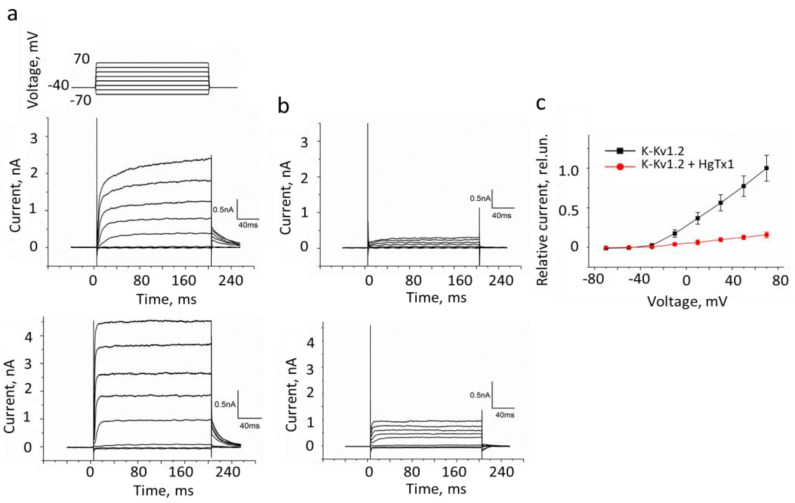
Currents in Neuro2a cells transfected with K-Kv1.2 that were measured by the patch-clamp technique using the whole-cell mode. (**a**,**b**) Representative traces of the currents in the absence (**a**) or in the presence of 2 nM HgTx1 (**b**). Two types of observed currents are shown (top and bottom graphs in panels (**a**,**b**). (**c**) The current-voltage dependence for the cells in the absence or in the presence of 2 nM HgTx1 (mean ± SEM, *n* = 10). The membrane potential increased from −70 to +70 mV with the 20 mV step each 20 s and returned to −40 mV. The duration of voltage pulses was 200 ms.

Measurements using a patch-clamp technique in the whole-cell configuration revealed two different types of outward non-inactivating currents in the Neuro2a cells expressing K-Kv1.2: with a fast and slow activation (Figure 5a). The maximum current amplitudes were 3–6 and 2–4 nA in cells with the fast and slowly activated channels, respectively (Figure 5a). The number of cells with slow-activated channels was less than 30% of the total number of the measured cells. As shown earlier, outward voltage-dependent ion currents in native Neuro2a cells are weak (100–200 pA) and have no relation to Kv1 channels [24,25]. These background currents contribute less than 10% to the currents measured in the K-Kv1.2-expressing cells.

Both fast and slowly activated K-Kv1.2 channels are almost completely blocked (84 ± 2%) by the application of 2 nM HgTx1 (Figure 5b,c). Residual currents include the contribution of the currents through the channels of Neuro2a cells.

The calculated current-voltage dependence shows that the threshold activation voltage of K-Kv1.2 channels in Neuro2a cells is about −30 mV, and the current amplitude depends linearly on the applied voltage in the −10–+70 mV range (Figure 5c).

As discussed in Section 4, the heterogeneity in activation rate is an intrinsic property of the native Kv1.2 channels and is not related to any modifications present in K-Kv1.2.

### 3.4. Genetically Encoded Fluorescent Ligand of Kv1.2 Channel

High activity of HgTx1 labeled with organic fluorophore (A-HgTx) toward the Kv1.2 channel has motivated us to develop the genetically encoded fluorescent (GEF) ligand of Kv1.2 on the basis of HgTx1. Recent studies show that GEF ligands can be a reliable alternative to classical fluorescently labeled ligands [35,48,49,50], but their design and applicability for imaging ion channels are still questionable. No GEF ligands active against the Kv1.2 channel have been reported.

In the GEF ligand designed by us (hereinafter abbreviated as HgTx-G), HgTx1 was fused at its C-terminus with an enhanced green fluorescent protein (eGFP) [51] via L3 polypeptide (GSGGSGGSGGTGGAGGATST) (Figure 1a). A construct containing maltose binding protein (MBP), His-tag, TEV protease cleavage site, and HgTx-G was produced in *E. coli* cells, purified with Ni-affinity chromatography, and cleaved with TEV protease (Figure 1b), finally providing 30 mg of fluorescent HgTx-G from 1 L of cell culture.

**Figure 6:**
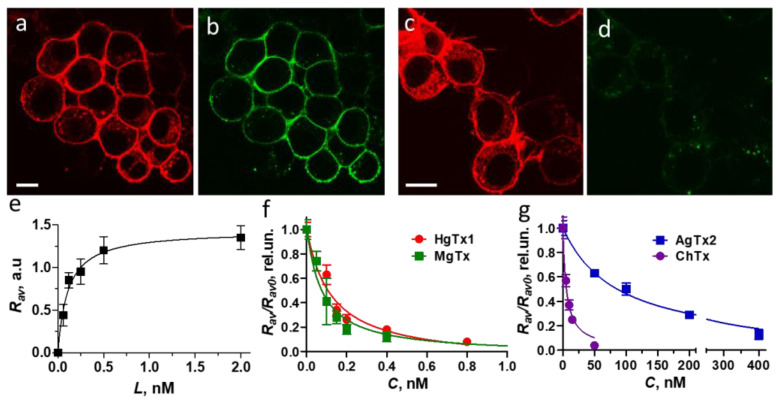
Interaction of HgTx-G with the Kv1.2 channel. (**a**–**c**) Confocal images of cells expressing K-Kv1.2 that were stained with HgTx-G (0.5 nM for 1 h) in the absence (**a**,**b**) or in the presence of competitor HgTx1 (1 nM) (**c**,**d**). Distributions of K-Kv1.2 (**a**,**c**) and HgTx-G (**b**,**d**) are shown in red and green, respectively. Bars are 15 µm. (**e**) Concentration dependence of HgTx-G binding to K-Kv1.2 that is presented in terms of the dependence of the *R_av_* parameter (see Section 2.8) on the concentration *L* of HgTx-G added to cells. (**f**,**g**) Concentration dependences of the competitive displacement of HgTx-G (0.5 nM) from the complexes with K-Kv1.2 by HgTx1 (**f**), MgTx (**f**), AgTx2 (**g**), or ChTx (**g**). *C*—concentration of the added competitor. Data (**e**–**g**) are averaged over three independent experiments and presented as mean ± SEM.

**Figure 7:**
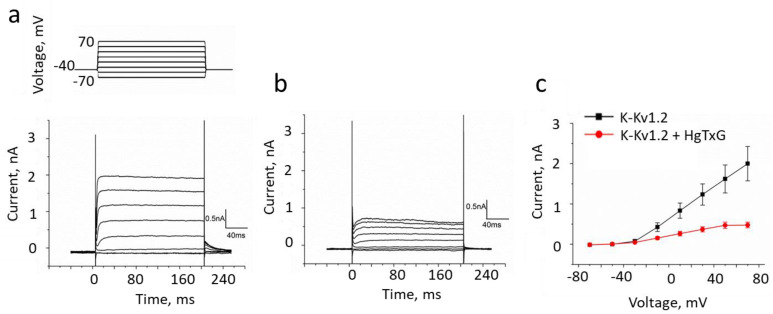
HgTx-G is a blocker of the Kv1.2 channel. (**a**,**b**) Representative traces of the currents in Neuro2a cells transfected with K-Kv1.2 that were measured by the patch-clamp technique using the whole-cell mode before (**a**) and after addition of 7.5 nM HgTx-G (**b**). (**c**) The current-voltage dependence for the cells before and after addition of 7.5 nM HgTx-G (mean ± SEM, *n* = 5). Inhibition of outward currents was 70 ± 5%. The membrane potential increased from −70 to +70 mV with the 20 mV step each 20 s and returned to −40 mV. The duration of voltage pulses was 200 ms.

A study of HgTx-G interaction with Neuro2a cells producing K-Kv1.2 revealed bright staining of the cell membrane with HgTx-G (Figure 6a,b), which was not observed when cells did not express the channel (Figure S1b). It should be noted that the excitation and emission fluorescence spectra of eGFP and mKate2 (a fluorescent tag used in K-Kv1.2) are well separated, which facilitates selective imaging of both fluorescent proteins when they simultaneously stain cells (Figure 6a,b). HgTx-G was displaced from the cell membrane by HgTx1 (Figure 6c,d), thus indicating that the binding sites of both ligands are situated at Kv1.2 and overlap. Binding of HgTx-G to Kv1.2 was observed at subnanomolar concentrations, saturated at ~0.5 nM, and characterized by a dissociation constant of 0.11 ± 0.05 nM (Figure 6e). Surprisingly, affinities of HgTx-G and A-HgTx to Kv1.2 are similar despite the presence of the bulk fluorescent tag in the former ligand. The electrophysiology study confirmed that the binding of HgTx-G is accompanied by blocking of the current through the Kv1.2 channel (Figure 7). HgTx-G also binds to the Kv1.1 and Kv1.3 channels and has dissociation constants of 0.4 ± 0.2 and 0.06 ± 0.03 nM, respectively (Figure S5).

HgTx-G bound to the Kv1.2 channel at the plasmalemma of Neuro2a cells does not penetrate into cells, does not affect the pattern of the channel distribution, and does not increase the channel internalization.

### 3.5. K-Kv1.2 and HgTx-G as Components of an Analytical System

Competition of a peptide pore blocker with HgTx-G for the binding to K-Kv1.2 channel (Figure 6c,d) allowed us to suppose that K-Kv1.2 and HgTx-G can be used as components of an analytical system for the identification of pore blockers among tested compounds as well as for the estimation of affinity of these blockers to Kv1.2 channel. Similar systems were previously developed by us on the basis of Kv1.1 and Kv1.3 channels [24,25].

To confirm the suitability of K-Kv1.2 and HgTx-G for such applications, concentration dependences of the displacement of HgTx-G from the complexes with K-Kv1.2 by several well-known peptide blockers were measured (Figure 6f,g) and analyzed using the previously approved formalism [24,25]. Apparent dissociation constants (*K_ap_*) of Kv1.2 complexes with peptides HgTx1, MgTx, ChTx, and AgTx2 were calculated to be 0.02 ± 0.01, 0.014 ± 0.010, 1.03 ± 0.11, and 21.5 ± 1.2 nM, respectively.

For comparison, 50% inhibition of the Rb^+^ flux through Kv1.2 channels in mammalian cells with depolarized membranes was observed at 0.14 nM HgTx1 [20]. According to our data, HgTx1 is sevenfold more active than reported earlier.

Fifty percent inhibition of the Rb^+^ flux through Kv1.2 occurred at 0.675 nM MgTx [20], while the patch-clamp measurements on human Kv1.2 channels in mammalian cells revealed the 50% decrease of current at 0.006 nM MgTx [52]. *K_ap_* of MgTx in our experiments is consistent with the last value.

Patch clamp measurements on rat Kv1.2 channels in mammalian cells revealed a 50% decrease in current at 10 nM ChTx [53]. Similarly, 9 nM ChTx inhibited 50% of the current through rat Kv1.2 in oocytes according to the two-electrode voltage clamp technique (TEVC) [54]. Tenfold higher activity of ChTx was observed in our experiments on human Kv1.2 channels.

AgTx2 was sevenfold weaker in activity on human Kv1.2 channels in our test system as compared to its activity on rat Kv1.2 channels measured on oocytes [54].

Definitely, different types of channels (human, rat), different “hosts” (oocytes, mammalian cells), and different principles of affinity measurement (patch clamp, Rb^+^ flux, TEVC, competitive displacement) may be reasons for the variations in the measured activities of the same peptide blocker. At the same time, it is generally agreed that HgTx1 and MgTx are highly active pore blockers, while AgTx2 and ChTx are moderately active blockers of the Kv1.2 channel.

### 3.6. Study of Ce Peptides

A group of Ce peptides found in the venom of the scorpion *Centruroides elegans* [55] includes Ce1 and Ce4 peptides, which, as reported, are moderately active blockers of the Kv1.3 channel [55], while Ce1 blocks also the Kv1.1 channel at nanomolar concentrations [25]. The structure of Ce1 and Ce4 peptides as well as their activity on the Kv1.2 channel were not studied yet. Ce1 and Ce4 are highly homologous to HgTx1 (Table 2), and molecular modeling using the Alfafold2 service predicted a similar secondary structure for these peptides, including three short antiparallel β-sheets and an α-helix stabilized by three disulfide bridges (Figure S6a). Reasonably, the predicted structure of HgTx1 is very similar to that determined by NMR spectroscopy (PDB entry 1HLY) [23]. MD experiments show that the models predicted by the Alfafold2 service are close to the energy equilibrated states, since root-mean-square deviation (RMSD) values calculated along MD trajectories for C_α_-atoms of cysteine residues are in ranges 0.2–0.5 Å for Ce1, 0.15–0.45 Å for Ce4, and 0.4–0.8 Å for HgTx1. Representative conformations of polypeptide chains of Ce4 and HgTx1 are very similar and differ slightly from that of Ce1 in the region of amino acid residues 9–12 (Figure S6b, Table 2). In this region, a short part of the 3–10 helix forms in Ce1 instead of the polypeptide turn in Ce4 and HgTx1.

In addition to the most representative conformation, alternative conformation was also obtained for each peptide. According to the MD data, the first five residues in the N-terminal region of the peptides under study exhibit significant conformational flexibility and may lose their β-sheet structure (Figure S6c). Similar conformational flexibility of the N-terminal region was previously revealed in MD experiments for OSK1 peptide blockers [56].

**Figure 8:**
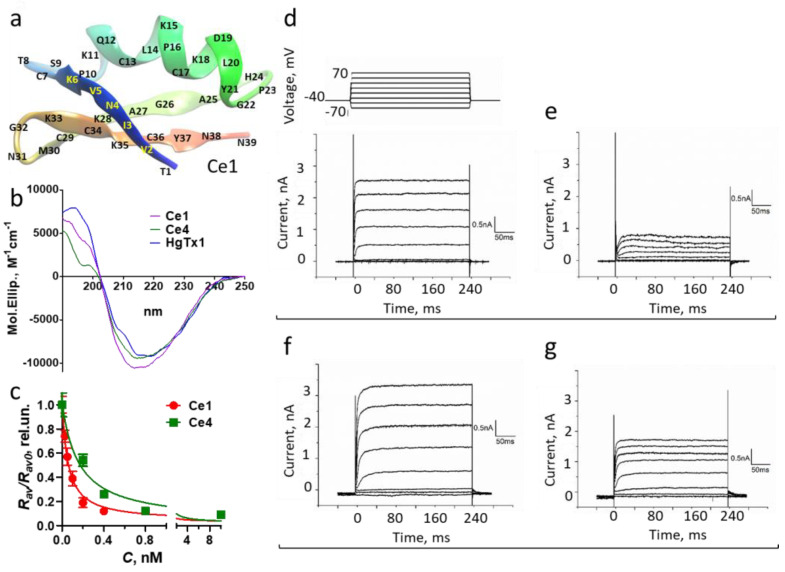
Structure of Ce peptides and their interactions with the K-Kv1.2 channel. (**a**) Structure of Ce1 peptide according to the AlfaFold2 prediction followed by MD relaxation. Structures of Ce4 and HgTx1 are similar to the structure of of Ce1, except for the slightly different conformation of the polypeptide chain in the region of amino acid residues 9–12 (Figure S6, Table 2). (**b**) CD spectra of Ce1, Ce4, and HgTx1 in a buffer containing 50 mM NaClO_4_, 12.5 mM Na_2_HPO_4_, and 12.5 mM NaH_2_PO_4_ (pH 7.5). (**c**) Concentration dependences of the competitive displacement of HgTx-G (0.5 nM) from the complexes with K-Kv1.2 by Ce1 and Ce4. The dependences of the *R_av_*/*R_av_*_0_ parameter (see Section 2.8) on the concentration *C* of the added competitor are presented. Data are averaged over three independent experiments and presented as mean ± SEM. (**d**–**g**) Representative traces of the currents in Neuro2a cells transfected with K-Kv1.2 that were measured by the patch-clamp technique using the whole-cell mode before (**d**,**f**) and after the addition of 10 nM Ce1 (**e**) or Ce4 (**g**).

To verify the Alfafold2 and MD predictions, the CD spectra of the peptides were measured (Figure 8b), and the content of the canonical types of secondary structures was calculated (Table S1). According to this calculation, the secondary structures of Ce1, Ce4, and HgTx1 are similar, while the observed differences in the CD spectra of the peptides are related to subtle alterations in the conformation of some residues that occur without essential changes in the structure of polypeptide scaffold.

Ce1 and Ce4 peptides were found to compete efficiently with HgTx-G for the binding to K-Kv1.2 (Figure 8c) and block current through the channel (Figure 8d–g). Calculations of the corresponding *K_ap_* revealed that Ce1 and Ce4 are high-affinity blockers of the Kv1.2 channel, and their activity is similar to that of HgTx1 and MgTx (Table 2).

According to data obtained on Kv1.1 and Kv1.3 channels previously [24,25] and in the current work (Figure S7) using similar analytical systems [24,25], peptides Ce1 and Ce4 are considerably more active on Kv1.2 channels as compared to Kv1.1 and Kv1.3 channels (Table 2). In the case of Ce4, factors of selectivity for Kv1.2 vs. Kv1.1 and for Kv1.2 vs. Kv1.3 are >10^4^ and 1000, respectively (Table 2).

### 3.7. Molecular Modeling of Peptide-Channel Complexes

To better understand the role of specific interactions between amino acid residues of the Kv1.2 channel and peptides Ce1, Ce4, and HgTx1 in tight binding, molecular modeling of the peptide-channel complexes was performed. For molecular modeling, a simplified channel model was created in which the cytoplasmic N- and C-terminal regions, which are not directly involved in interactions with peptides, were removed to facilitate calculations. Two channel models (amino acid residues 141–430), which differ in residue 371 (either serine or threonine), were obtained as described in Section 2.9. According to the MD data, the structure of the channel was not altered by the S371T mutation either in the immediate vicinity of the mutated amino acid or in other parts of the channel (Figure S8). Some deviations in the structure of the polypeptide chains observed in the channel models, especially on the extracellular side of the membrane, are within the range of “thermal fluctuations”, which are also seen in different frames of MD trajectories.

The channel model and the representative models of peptides were subjected to the docking procedure. Based on the data from electrophysiological studies, those docking solutions were selected for further experiments where the side chain of one of the amino acid residues of the peptide occluded the channel pore. The selected models of the complexes were subjected to molecular dynamics (MD) simulations during 100 ns, and the interactions between the amino acid residues of the channel and peptides were analyzed.

**Figure 9:**
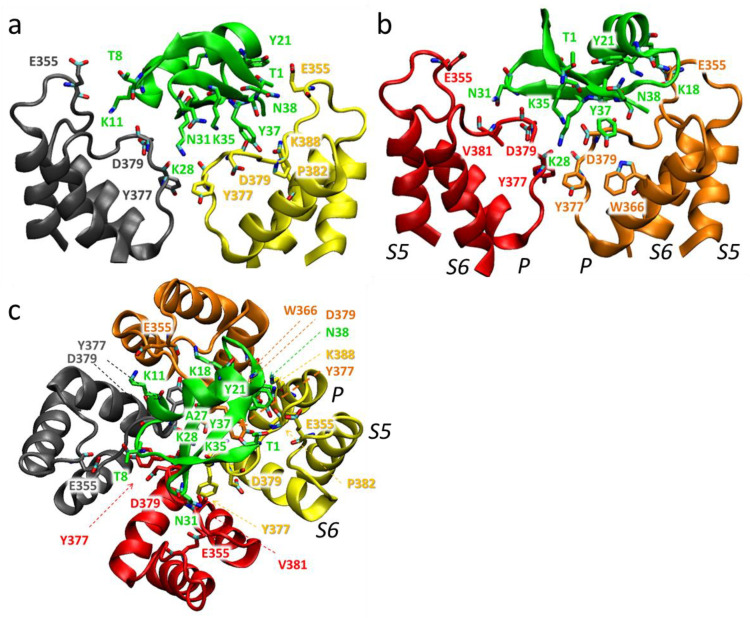
Structural model of the Kv1.2-Ce1 complex: side (**a**,**b**) and top (extracellular) (**c**) views. Membranous and extracellular parts of the pore domain are shown. Backbones of the channel α-subunits are shown in grey, yellow, red, and brown ribbons and solid lines. The Ce1 backbone is shown in green ribbon and a solid line. For clarity, the back and front α-subunits of the channel are not visible in the side views (**a**,**b**). The interacting residues, which according to MD data have an average frequency of contacts >20%, are marked and shown in a stick presentation. In the interacting side chains of peptide residues, nitrogen, and oxygen atoms are depicted in blue and red, respectively*. P, S5*, *and S6* are a pore helix (P) and transmembrane S5 and S6 helixes of the α-subunit, respectively.

**Figure 10:**
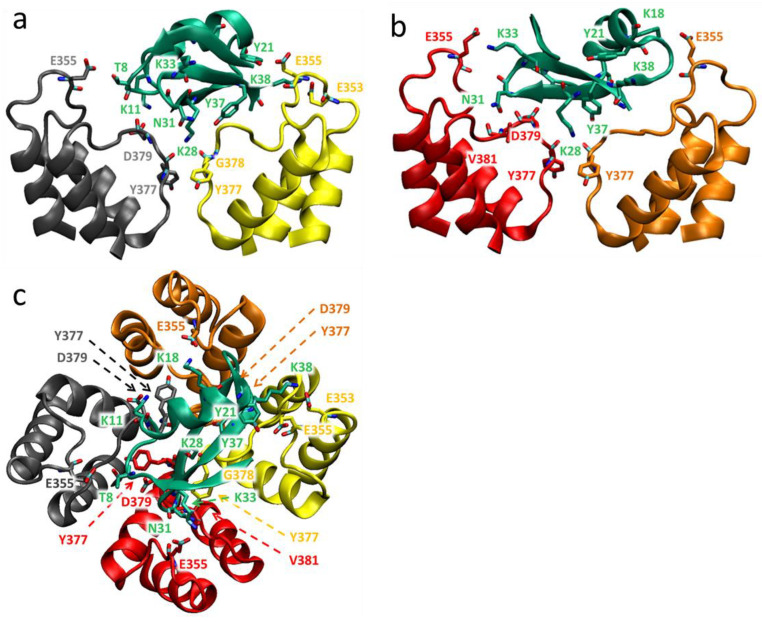
Structural model of the Kv1.2-Ce4 complex: side (**a**,**b**) and top (extracellular) (**c**) views. Membranous and extracellular parts of the pore domain are shown. Backbones of the channel α-subunits are shown in grey, yellow, red, and brown ribbons and solid lines. The Ce4 backbone is shown in green ribbon and a solid line. For clarity, the back and front α-subunits of the channel are not visible in the side views (**a**,**b**). The interacting residues, which according to MD data have an average frequency of contacts >20%, are marked and shown in a stick presentation. In the interacting side chains of peptide residues, nitrogen, and oxygen atoms are depicted in blue and red, respectively.

**Figure 11:**
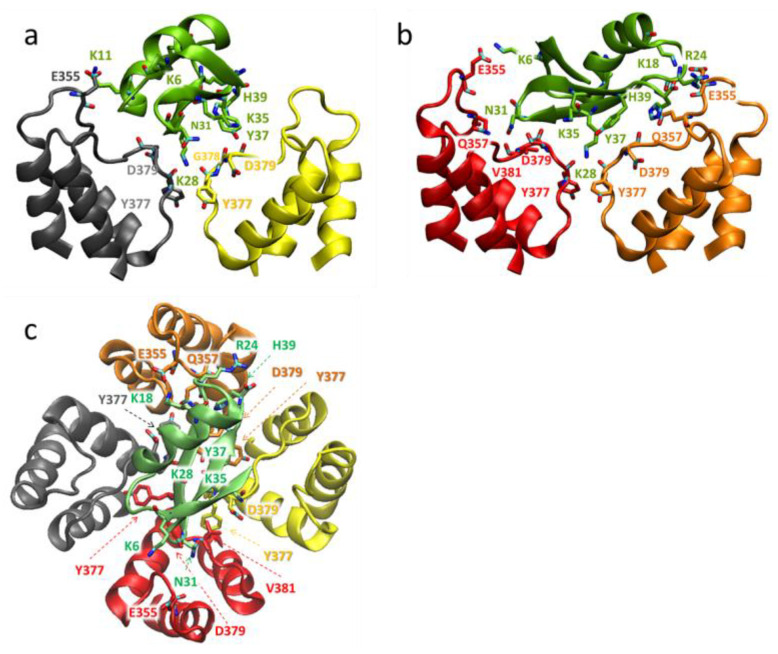
Structural model of the Kv1.2-HgTx1 complex: side (**a**,**b**) and top (extracellular) (**c**) views. Membranous and extracellular parts of the pore domain are shown. Backbones of the channel α-subunits are shown in grey, yellow, red, and brown ribbons and solid lines. The HgTx1 backbone is shown in a green ribbon and a solid line. For clarity, the back and front α-subunits of the channel are not visible in the side views (**a**,**b**). The interacting residues, which according to MD data have an average frequency of contacts >20%, are marked and shown in a stick presentation. In the interacting side chains of peptide residues, nitrogen, and oxygen atoms are depicted in blue and red, respectively.

**Figure 12:**
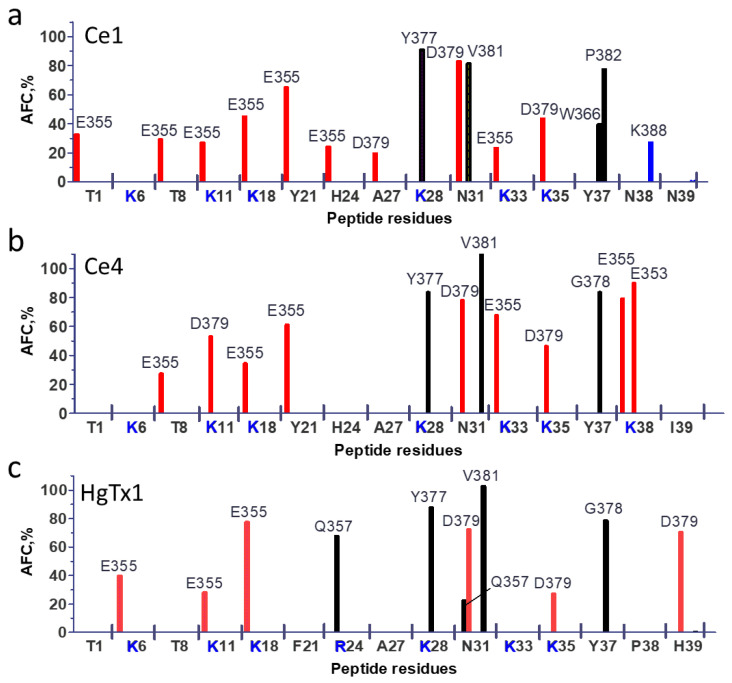
Average frequencies of contacts (AFC) between the residues of the peptide and channel, which participate in electrostatic interactions and/or form hydrogen bonds, according to MD data. Data are shown for Ce1 (**a**), Ce4 (**b**) and HgTx1 (**c**). Data for negatively charged, positively charged and uncharged residues are shown in red, blue and black, respectively. Contacts that have AFC > 20% are shown.

In the models of complexes obtained, peptides bind to the extracellular side of the pore domain of the channel, specifically at the plateau region formed by the α-subunits around the ion-conducting pore (Figure 9, Figure 10 and Figure 11). This plateau is surrounded at its edges by so-called turrets formed by polypeptide loops connecting the S5 transmembrane helix with a short pore helix of each α-subunit (Figure 9). The secondary structure of the peptides is not significantly altered upon binding. The orientation of the bound peptides with respect to the channel is similar: the second and third β-sheets are in contact with the plateau, while the helical fragment is largely exposed to the solution (Figure 9, Figure 10 and Figure 11). The ion pore is occluded by the side chain of the K28 residue of each peptide, which forms hydrogen bonds with the Y377 residues of α-subunits situated in the outer vestibule of the ion pore. A comparison of the superimposed models of peptide-channel complexes revealed that Ce1 and HgTx1 are shifted slightly relative to each other in the complexes, while Ce4 appeared to be slightly rotated relative to Ce1 and HgTx1 (Figure S9). This rotation is likely caused by the presence of lysine at position 38 in the Ce4 peptide. The K38 residue strongly interacts with the E353 and E355 residues of the channel (Figure 10 and Figure 12b), stabilizing the turned orientation of the peptide within the binding site.

In the Kv1.2-Ce1 complex, thirteen residues of Ce1 participate in electrostatic and/or hydrogen bonding to 16 residues of α-subunits (Figure 9 and Figure 12a). In the Kv1.2-Ce4 complex, electrostatic interactions and/or hydrogen bonds form between ten residues of Ce4 and 15 residues of α-subunits (Figure 10 and Figure 12b). In the Kv1.2-HgTx1 complex, 14 residues of α-subunits are involved in electrostatic and/or hydrogen bonding to nine residues of HgTx1 (Figure 11 and Figure 12c). This suggests that high-affinity binding is achieved through multipoint interactions of these peptides with Kv1.2.

Four α-subunits participate in interactions with peptides to varying degrees. Three α-subunits interact with Ce1 more strongly (5, 4, and 4 bonds are formed) compared to the fourth one (3 bonds) (Figure 9). In the Ce4-Kv1.2 complex, two α-subunits form three bonds each, while the other two produce five and four bonds (Figure 10). Participation of all α-subunits in the binding of HgTx1 is more uniform, with 4, 3, 4, and 3 bonds formed (Figure 11). The most active participation in the interactions with peptides is taken by amino acid residues E355 and Y377: each α-subunit delegates E355 and Y377 to peptide binding (with the exception of E355 from one α-subunit in the Kv1.2-HgTx1 complex). In addition, the D379 residue of each α-subunit interacts with Ce1 and HgTx1. Other amino acid residues of the channel that are involved in peptide binding belong to one or, in the case of the Q357 residue, two α-subunits. It should be noted that the bond between V381 of the channel and the Y37 invariant residue of peptides occurs in all three complexes.

A specific feature of the interactions between peptides and the channel is that the largest number of strong bonds is formed in the region of the last nine amino acid residues of the Ce1, Ce4, and HgTx1 peptides: 7 of 15, 7 of 12, and 6 of 11 bonds, respectively (Figure 11). Both invariant and some variable amino acid residues of the Ce1, Ce4, and HgTx1 peptides contribute to the channel binding (Table 2, Figure 9, Figure 10, Figure 11 and Figure 12). Among the invariant amino acid residues in the Ce1, Ce4, and HgTx1 peptides, the interactions of the K11, K28, N31, K35, and Y37 residues with the channel seem to provide a common basis for complex stabilization. The interactions of the channel with other invariant amino acid residues, such as T1, K6, T8, K18, A27, and K33, vary among peptides. These peptide-specific interactions are supposed to compensate for the possible negative effect of some variable residues on the complex stability.

Six of the ten variable residues do not interact with the channel. Among the other variable residues, Y21 in Ce1 and Ce4 interacts with the channel in contrast to the F21 residue in HgTx1. According to docking simulations of HgTx1 complexes with the Kv1.1, Kv1.2, and Kv1.3 channels, the R24 residue of HgTx1 plays a role in the complex stabilization due to the formation of the salt bridges with the channel amino acid residues [57]. In our MD model, the cationic R24 residue in HgTx1 forms not an ionic but a hydrogen bond in complex with the Kv1.2 channel, and its replacement with histidine in peptides Ce1 and Ce4 seems to have a weak effect on the stability of the peptide complexes with the channel. In the complex Ce1-Kv1.2, the H24 residue forms a hydrogen bond as well, while the absence of the H24 interaction with the channel in the Ce4-Kv1.2 complex is probably compensated by other interactions. As discussed above, the K38 residue in Ce4 is strongly involved in the complex formation. The N38 residue in Ce1 also interacts with the channel, as opposed to P38 in HgTx1. Regarding the C-terminal variable residue, only the histidine residue in the HgTx1 peptide interacts with Kv1.2 in contrast to the asparagine and isoleucine residues in the Ce1 and Ce4 peptides, respectively.

## 4. Discussion

We report that two fluorescently labeled derivatives of HgTx1, namely, A-HgTx and HgTx-G, are high-affinity ligands of the Kv1.2 channel that retain the pore-blocking ability of parent HgTx1 and have *K_d_* of 80 ± 20 and 110 ± 50 pM, respectively (Figure 2 and Figure 6).

A-HgTx is fourfold less active on Kv1.2 than HgTx1 (Figure 6) but qualitatively preserves the pharmacological profile of HgTx1, being also a high-affinity ligand of channels Kv1.1 (*K_d_* = 700 ± 200 pM [25]) and Kv1.3 (*K_d_* = 300 ± 130 pM [24]). Previously, labeling of HgTx1 with fluorophores Cy3, Cy5, Alexa 488, Alexa 546, Alexa 594, Alexa 660, and Alexa 680 was performed at the cysteine residue introduced instead of alanine 19 [23]. It was demonstrated that these fluorescent derivatives retain the pharmacological profile of HgTx1 and have very high activity for native Kv1.1/Kv1.2 channels in rat brain synaptosomal membranes, but the *K_d_* values for their complexes with Kv1.2 or Kv1.1 channels were not measured. New (Figure 6) and previously obtained [24,25] data suggest that labeling HgTx1 with an organic dye at the N-terminus produces a highly active fluorescent probe for Kv1.2, Kv1.1, and Kv1.3 potassium channels.

HgTx1 labeled with eGFP at the C-terminus is the first genetically encoded fluorescent ligand having high activity for the Kv1.2 channel. It is also highly active on the Kv1.1 and Kv1.3 channels (*K_d_* values are 400 ± 200 and 60 ± 30 pM, respectively (Figure S5)), thus having a pharmacological profile that is qualitatively similar to that of HgTx1. HgTx-G activity is similar to that of A-HgTx on the Kv1.2 channel, but it is slightly higher on Kv1.1 and five times higher on Kv1.3. HgTx-G production is facilitated due to the high yield and lack of a refolding step in the manufacturing process. The only drawback is that the fluorescence intensity of eGFP is about half that of the Atto 488 dye.

As shown, A-HgTx and HgTx-G are useful for imaging recombinant Kv1.2 channels in cells and for studying natural and artificial peptide blockers of these channels (Figure 2 and Figure 6).

Although the Kv1.2 channel is widely presented in the membrane of cells of the CNS [58], studies of K-Kv1.2wt have shown that, in Neuro2a cells, the dominant part of the Kv1.2 α-subunits was retained in the cytoplasm, while the membrane traffic of the Kv1.2 channel was very low (Figure 2). A relatively low level of the recombinant Kv1.2 channel was also observed on the surface of other types of cells [44,59]. It was reported that auxiliary Kvβ1 and Kvβ2 subunits, which co-assemble with Kv1 channels, provide membrane trafficking of Kv1.2 channels in mammalian cells, as well as axonal targeting in neurons [59,60,61]. The imbalance between the high expression level of the recombinant Kv1.2 channels and the low natural level of Kvβ subunit synthesis is suggested to restrict the membrane targeting of the recombinant channels [59].

It has been demonstrated that the natural suppressors of the Kv1.2 membrane trafficking are S371 in the deep pore region [32] and N469 in the C-terminal region of the channel [44,45]. The type of amino acids at positions 371 and 381 is important for glycosylation, which has been suggested to enhance the membrane presentation of Kv1.2 channels [46,47]. In the mechanism of channel trafficking to plasmalemma, the type of amino acid at position 469 affects the transfer of channels from ER to the Golgi apparatus [45]. Our results (Figure 2) confirm previous observations that the S371T mutation, in the presence of the V381 residue, enhances the transport of Kv1.2 to the plasmalemma [44]. We report also that an additional membrane-targeting mutation, N469L, provides no synergistic effect with mutation S371T in the Kv1.2 channel (Figure 2), even though these two mutations affect different stages of trafficking [45].

It should be noted that the low level of channel accumulation in lysosomes (Figure S3) indicates the correct incorporation of the synthesized subunit into ER and the absence of aggregation despite the high level of synthesis of the recombinant channel [62].

The low localization of the channel in the endosomes of Neuro2a cells (Figure S2) suggests that it is not significantly involved in endocytosis, which in the case of Kv1.2 is regulated by channel phosphorylation [63]. 

Since the potential ability of recombinant Kv1 channels to integrate into the mitochondrial membrane was discussed [64], we report that the recombinant Kv1.2 channels are not localized in the mitochondria in Neuro2a cells (Figure S4).

Two electrophysiologically different types of K-Kv1.2 channels were observed in Neuro2a cells: fast and slowly activated channels (Figure 5). The inconstant character of the Kv1.2 channel activation is well known. Fast-activated Kv1.2 channels are a more typical case. They have been detected in the transfected *Xenopus laevis* oocytes [65,66] and in mammalian cell lines [67,68]. Slow activation of Kv1.2 channels was described for mouse fibroblast cells B82, which stably expressed rat Kv1.2 channels [69]. The presence of the Kv1.2 channels with both types of activation was also found in mammalian cells [70]. Thorough studies revealed that the variability in the channel activation is mediated by the highly specific threonine residue in the S2,S3 linker of Kv1.2 channels [70]. Recently, it was confirmed that the variability of Kv1.2 activation is the innate property of the channel [71]. Thus, the electrical properties of the K-Kv1.2 channel with the S371T mutation and mKate2 fluorescent protein at the N-terminus are similar to those of the native Kv1.2 channel.

Fluorescence, a high level of membrane expression of K-Kv1.2, and a high affinity of the HgTx-G ligand for the Kv1.2 channel significantly facilitate the use of K-Kv1.2, HgTx-G, and laser scanning fluorescence microscopy to qualitatively discriminate and quantitatively study peptide blockers of Kv1.2 using a previously developed methodological approach and formalism [24].

Such studies conducted on living mammalian cells under physiological conditions are more relevant to assess the applicability of blockers, including fluorescent ligands, for neuroscience and better meet the general requirements for preclinical studies of drug candidates.

Ce1, Ce4, and HgTx1 are high-affinity homologous ligands of the Kv1.2 channel (Table 2). Considering MD data (Figure 9, Figure 10, Figure 11 and Figure 12), we conclude that the stability of the complexes between these peptides and the channel is provided by a complex network of interactions between amino acid residues of peptides and the channel. From the channel side, the common basis for high-affinity binding of the Ce1, Ce4, and HgTx1 peptides is formed by the E355, Y377, D379, and V381 residues of the Kv1.2 channel, while some other channel residues contribute to the interactions depending on the particular peptide. From the peptide side, the interactions of invariant residues K11, K28, N31, K35, and Y37 of these peptides provide the basis for strong binding, while the variable residues do not cause critical stabilization or destabilization of the complexes. It is possible that some substitutions of variable residues are unfavorable; however, their effect is compensated for by interactions of other invariant and variable residues with the channel.

According to the conducted studies (Figure 8 and Figure S7, Table 2), peptides Ce1 and Ce4 extend the list of selective blockers of the Kv1.2 channel. Ce4 is comparable in activity but slightly inferior in selectivity to the engineered peptide ChTX(M29I), the most active and selective blocker of the Kv1.2 channel [31]. Ce4 is similar in activity but surpasses slightly in selectivity another remarkable selective blocker of the Kv1.2 channel, the peptide CboK7 from the *Centruroides bonito* scorpion venom [30]. Ce4 exceeds both in activity and selectivity other highly selective blockers of Kv1.2, including the peptides MeKTx11-1 [29], urotoxin [72], and mesomartoxin [28]. Ce1 is 10 times inferior to peptide Ce4 in terms of selectivity to Kv1.2 vs. Kv1.1 (Table 2). Homology between Ce4 (Ce1) and ChTX(M29I), MeKTx11-1, urotoxin, or mesomartoxin is low. In contrast, Ce1 is 90% identical to the CboK7 peptide, differing in four substitutions: V2F, A27E, N38K, and N39V. The identity of Ce4 and CboK7 is 82%, and differences include substitutions I2F, L15K, E19D, I20L, I23P, A27E, and I39V.

According to our models, the amino acid residues at positions 2, 15, 19, 20, 23, and 39 do not interact with the Kv1.2 channel in complexes formed by Ce1 and Ce4 peptides (Figure 9,Figure 10 and Figure 12). That is why, we suppose, substitutions at these positions in CboK7 do not significantly affect the stability of the CboK7-Kv1.2 complex compared to the Ce4-Kv1.2 and Ce1-Kv1.2 complexes. The presence of K38 in Ce4 removes the A27 residue from interaction with the channel, in contrast to the Ce1-Kv1.2 complex (Figure 12a,b). CboK7 also contains lysine at position 38, which may explain the lack of an essential effect of the A27E substitution on the stability of the CboK7-Kv1.2 complex compared to the Ce4-Kv1.2 complex.

The analysis of the interaction mechanism that determines the selectivity of peptides Ce1, Ce4, and CboK7 toward the Kv1.2 channel is complicated by the complex multipoint nature of interactions between these peptides and the channel. This analysis, which employs methods of molecular modeling and site-directed mutagenesis, is currently in progress. A comparison of the amino acid sequences of the Kv1.1-Kv1.3 channels in the peptide binding region (Table 3) suggests that the selectivity of interaction may be associated with the variable residues at positions 354, 355, 357, and 381 (numbering according to the sequence of Kv1.2). From the peptide side, certain residues that differ in HgTx1 from those in Ce1, Ce4, and CboK7 may be involved in determining the selectivity of the interaction. The residues at positions 4, 15, 21, 24, 38, and 39 are the most likely candidates.

## 5. Conclusions

The properties of A-HgTx described previously [24,25] and in this work, as well as the properties of genetically encoded HgTx-G, developed by us, allow one to conclude that these channel blockers are high-affinity fluorescent probes for imaging Kv1.1, Kv1.2, and Kv1.3 channels in cells and for studying natural and artificial peptide blockers of these channels. To support further applications of HgTx-G, we are currently investigating its efficacy and potency as a blocker of Kv1.1, Kv1.2, and Kv1.3 channels. Based on the data obtained, the recombinant peptides Ce1 and Ce4 derived from the venom of the scorpion C. elegans are highly effective blockers of the Kv1.2 channel. The peptide-channel complexes were concluded to be stabilized by interactions between the invariant residues K11, K28, N31, K35, and Y37 of these peptides and E355, Y377, D379, and V381 of the Kv1.2 channel. Peptides Ce1 and Ce4 are highly selective blockers of the Kv1.2 channel, compared to the Kv1.1 and Kv1.3 channels.

Extended studies of Ce1 and Ce4 are currently underway to gain a deeper understanding of their properties and selectivity mechanisms. These studies include site-directed mutagenesis of these peptides and Kv1 channels complemented with molecular modeling experiments. The efficacy and potency of Ce1 and Ce4 as Kv1.2 channel blockers will be further clarified in an electrophysiological study of the response of current through Kv1.2 to varying concentrations of these peptides.

Peptides Ce1 and Ce4 are supposed to find application in the studies of the native Kv1.2-mediated currents in neurons and can be also used to construct Kv1.2 channel-specific fluorescently labeled ligands.

## Figures and Tables

**Table 1:** Oligonucleotide primers.

Notation	Nucleotide Sequence ^1^
Kcna2-f1	5′-TTCTCAGATCT**ATG**ACAGTGGCCACCGGAGACCCA-3′
Kcna2-r1	5′-TTCTCAAGC**TT****A**GACATCAGTTAACATTTTGGTAATATTC-3′
Kcna2m1-f1	*5′-GGCAGTCGTC**ACC**ATGACAACTG* TAGGCTATG-3′
Kcna2m1-r1	*5′-CAGTTGTCAT**GGT**GACGACTGCC* CACCAGAAGG-3′
Kcna2m2-f1	5′-*GTGTAAATAACAGT**CTG**GAGGACTTTAG* AGAGGAAAAC-3′
Kcna2m2-r1	*5′-CTAAAGTCCTC**CAG**ACTGTTATTTACAC* CCTCCTGGAT-3′
Hg-f1	5′-TTCTCGGTACCGAAAACCTGTATTTTCAG-3′
Hg-r1	5′-TTCTCGGATCCATGCGGATAACATTTGCATT-3′

^1^ Sites of restriction enzymes are underlined. Start and stop codons (ATG and TTA) in forward Kcna2-f1 and reverse Kcna2-r1 primers; codons for T371 (ACC and GGT) in forward Kcna2m1-f1 and reverse Kcna2m1-r1 primers; as well as codons for L469 (CTG and CAG) in forward Kcna2m2-f1 and reverse Kcna2m2-r1 primers are marked in bold. Overlapping sequences are shown in italics.

**Table 2:** Sequences of Ce1, Ce4, HgTx1, and MgTx peptides and dissociation constants (*K_ap_*, nM) of their complexes with Kv1.2, Kv1.1, and Kv1.3 channels.

Peptide	Amino Acid Sequence	Identity, %		*K_ap_,* nM			
		HgTx1	MgTx		Kv1.2	Kv1.1	Kv1.3
Ce1	1 10 20 30	77	77		0.010 ± 0.002	11 ± 5 ^$^	13 ± 8 ^&^
	**TVINVKCTSPKQCLKPCKDLYGPHAGAKCMNGKCKCYNN** * CEEEEECCCGGGHHHHHHHHHTTTEEEEEETTEEEEEEC						
Ce4	**TIINVKCTSPKQCLLPCKEIYGIHAGAKCMNGKCKCYKI** CEEEEECCTTTTHHHHHHHHHTTTEEEEEETTEEEEEEC	77	80		0.030 ± 0.010	>300 ^&^	30 ± 10 ^&^
HgTx1	**TVIDVKCTSPKQCLPPCKAQFGIRAGAKCMNGKCKCYPH** CEEEEECCTTTTHHHHHHHHHTTTTEEEEETTEEEEECC	100	90		0.020 ± 0.010	0.03 ± 0.02 ^$^	0.2 ± 0.1 ^#^
MgTx	**TIINVKCTSPKQCLPPCKAQFGQSAGAKCMNGKCKCYPH** CEEEEETTTGGGGTTGGGTTTTTTEEECTBTTEEEEEEC	90	100		0.014 ± 0.010	0.3 ± 0.2 ^$^	1.4 ± 0.7 ^&^

* Bold letters—amino acid residues. Plain letters—assignments of amino acid residues to particular types of secondary structure (T-turn, E-extended conformation (β-structure), B—isolated bridge, H—α helix, G—3–10 helix, C-coil) according to the structures of Ce1, Ce4, and HgTx1 presented in Figure S6b and the NMR structure of MgTx (PDB entry 1MTX). Variable residues are underlined; ^$^ [25]; ^&^ Supplementary materials, Figure S7; ^#^ [24].

**Table 3:** Amino acid sequences of the P-loop region of Kv1.1-Kv1.3 channels.

Channel	Amino Acid Sequence of the Peptide Binding Site *	
Kv1.1	346	386
	FAEAEEAESHFSSIPDAFWWAVVSMTTVGYGDMYPVTIGGK	
Kv1.2	348	388
	FAEADERESQFPSIPDAFWWAVVSMTTVGYGDMVPTTIGGK	
Kv1.3	418	458
	FAEADDPTSGFSSIPDAFWWAVVTMTTVGYGDMHPVTIGGK	

* Variable residues are underlined.

## Data Availability

The data presented in this study are available on request from the corresponding author. The data are not publicly available due to local regulations.

## Supplementary Materials

The following supporting information can be downloaded at: https://www.mdpi.com/article/10.3390/cells13242096/s1, Figure S1: Control measurements of fluorescence from Neuro 2a cells; Figure S2: Analysis of localization of K-Kv1.2wt, K-Kv1.2 and K-Kv1.2m2 in lysosomes of Neuro 2a cells; Figure S3: Analysis of localization of K-Kv1.2wt, K-Kv1.2 and K-Kv1.2m2 in endosomes of Neuro 2a cells; Figure S4: Analysis of localization of K-Kv1.2wt, K-Kv1.2 and K-Kv1.2m2 in mitochondria of Neuro 2a cells; Figure S5: Concentration dependences of HgTx-G binding to recombinant Kv1.1 and Kv1.3 channels engineered and expressed in Neuro2a cells as described earlier [24,25]; Figure S6: Structures of Ce1, Ce4 and HgTx1 predicted by AlfaFold2 (a) and further subjected to molecular dynamics relaxation (b,c); Figure S7: Competitive displacement of A-HgTx (2 nM) from the complexes with K-Kv1.1 (a,b) and K-Kv1.3 (c) by Ce1, Ce4, and MgTx peptides; Figure S8: Side view of the merged models of the Kv1.2 channel (green color) and Kv1.2(S371T) channel (different subunits are shown in different colors); Figure S9: Merged models of the complexes between Kv1.2 channel and peptides Ce 1, Ce4 and HgTx1 (view from the external surface of the membrane); Table S1: Content of secondary structures in the studied peptides (%) according to the CD analysis.
