# Supplementary material for: New High-Affinity Peptide Ligands for Kv1.2 Channel: Selective Blockers and Fluorescent Probes
Source: Cells. 2024 Dec 18;13(24):2096. doi: 10.3390/cells13242096 (PMC11674118; doi:10.3390/cells13242096)
Supplement: Supplementary file 1 [file cells-13-02096-s001.zip › cells-3306535-supplementary.pdf]

Supplementary materials to

New high-affinity peptide ligands for Kv1.2 channel: selective blockers and fluorescent probes

Anastasia A. Ignatova, Elena V. Kryukova, Valery N. Novoseletsky, Oleg V. Kazakov, Nikita A. Orlov, Varvara N. Korabeynikova, Maria V. Larina, Arkady F. Fradkov, Sergey A. Yakimov, Mikhail P. Kirpichnikov, Alexey V. Feofanov and Oksana V. Nekrasova

Control measurements of fluorescence from Neuro 2a cells

Experiments were performed as described in the *Material and Methods* section.

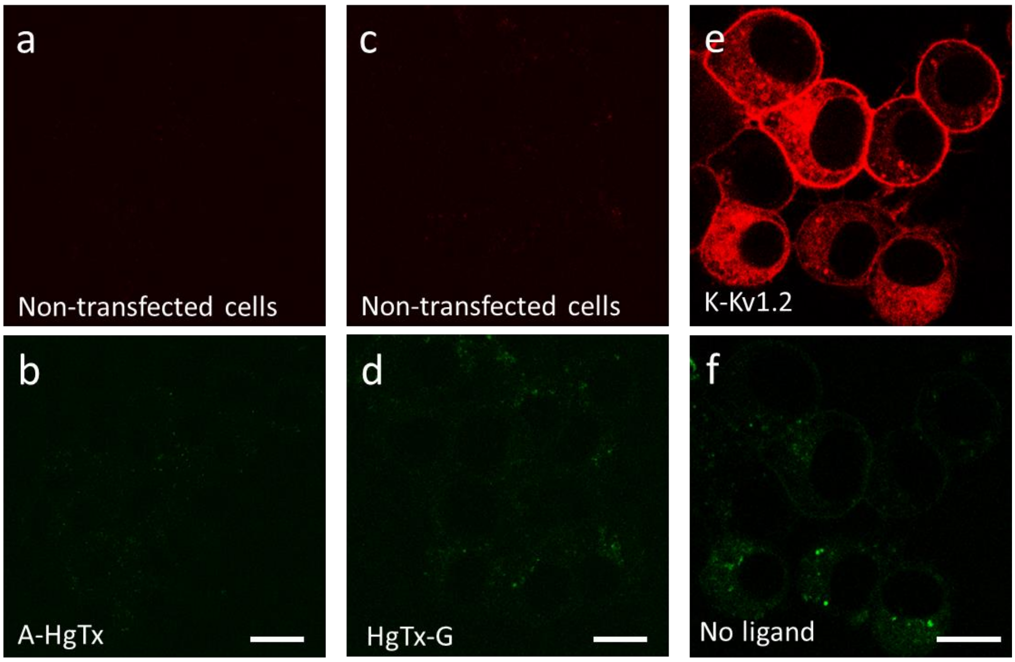

Figure S1. Control measurements of fluorescence from Neuro 2a cells. (a-d) Non-transfected cells were stained with 2 nM A-HgTx (a, b) or 2 nM HgTx-G (c, d) for 60 min. (e,f) Cells were transfected with K-Kv1.2 and imaged without staining with any fluorescent ligand. Distributions of fluorescence were measured using confocal microscopy: (a,c,e) – excitation at 561 nm, detection at 650- 700 nm; (b,d,f) – excitation at 488 nm, detection at 498-535 nm. Bar is 20 µm.

Analysis of localization of K-Kv1.2wt, K-Kv1.2 and K-Kv1.2m2 channels in lysosomes, endosomes and mitochondria of Neuro2a cells

Experiments were performed as described in the *Material and Methods* section.

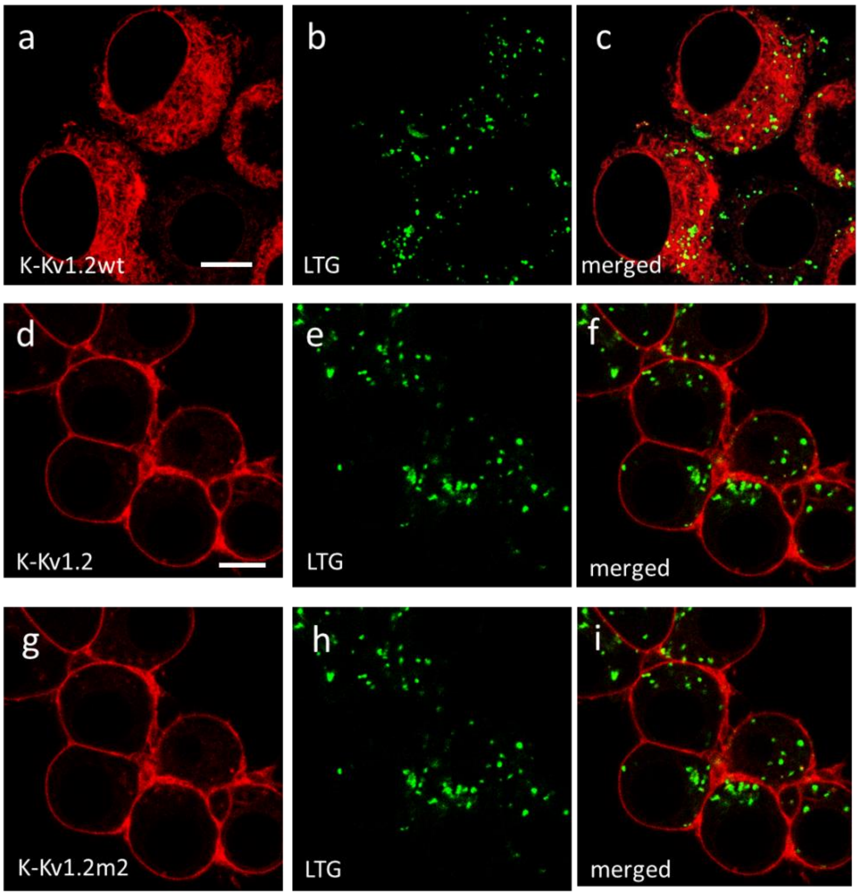

Figure S2. Analysis of localization of K-Kv1.2wt, K-Kv1.2 and K-Kv1.2m2 in lysosomes of Neuro 2a cells. (a,d,g) Confocal images of a distribution of K-Kv1.2wt (a), K-Kv1.2 (d) and K-Kv1.2m2 (g) in cells. (b,e,h) Confocal images of a distribution of the fluorescent marker of lysosomes (LTG) in cells. (c,f,i) Merged images of LTG and Kv1.2 channels. In the case of the co-localization of Kv1.2 and LTG in lysosomes, an appearance of yellow color is expected. Bar is 20 µm.

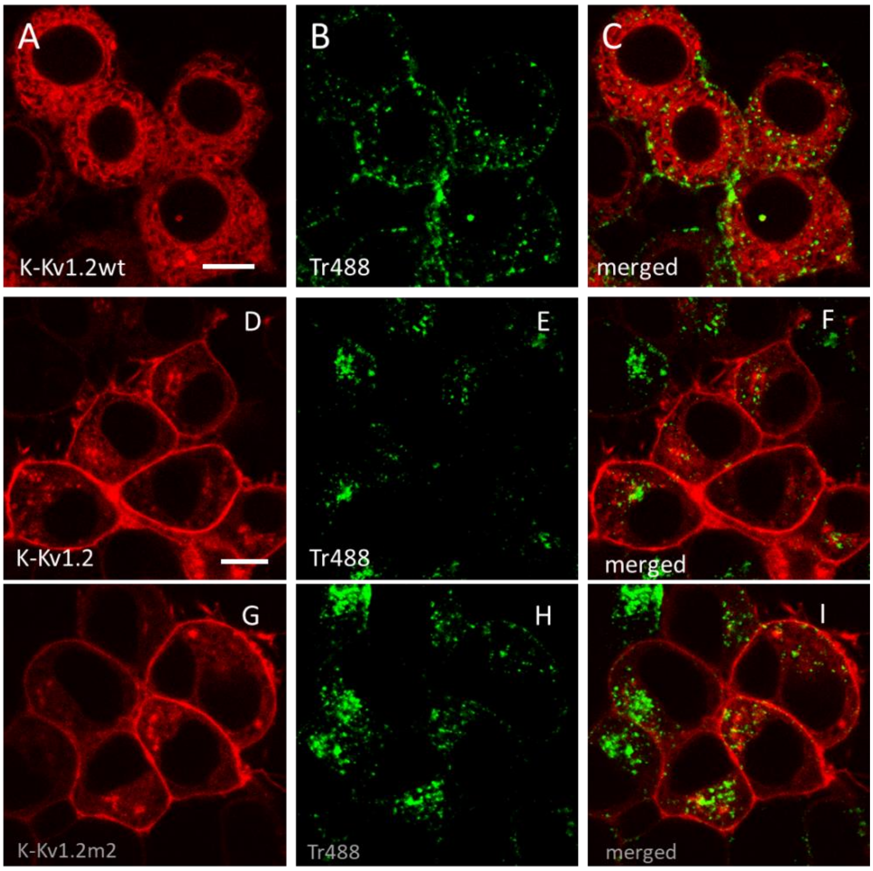

Figure S3. Analysis of localization of K-Kv1.2wt, K-Kv1.2 and K-Kv1.2m2 in endosomes of Neuro 2a cells. (a,d,g) Confocal images of a distribution of K-Kv1.2wt (a), K-Kv1.2 (d) and K-Kv1.2m2 (g) in cells. (b,e,h) Confocal images of a distribution of the fluorescent marker of endosomes (Tr488) in cells. (c,f,i) Merged images of Tr488 and Kv1.2 channels. In the case of the co-localization of Kv1.2 and Tr488 in endosomes, an appearance of yellow color is expected. Bar is 20 µm.

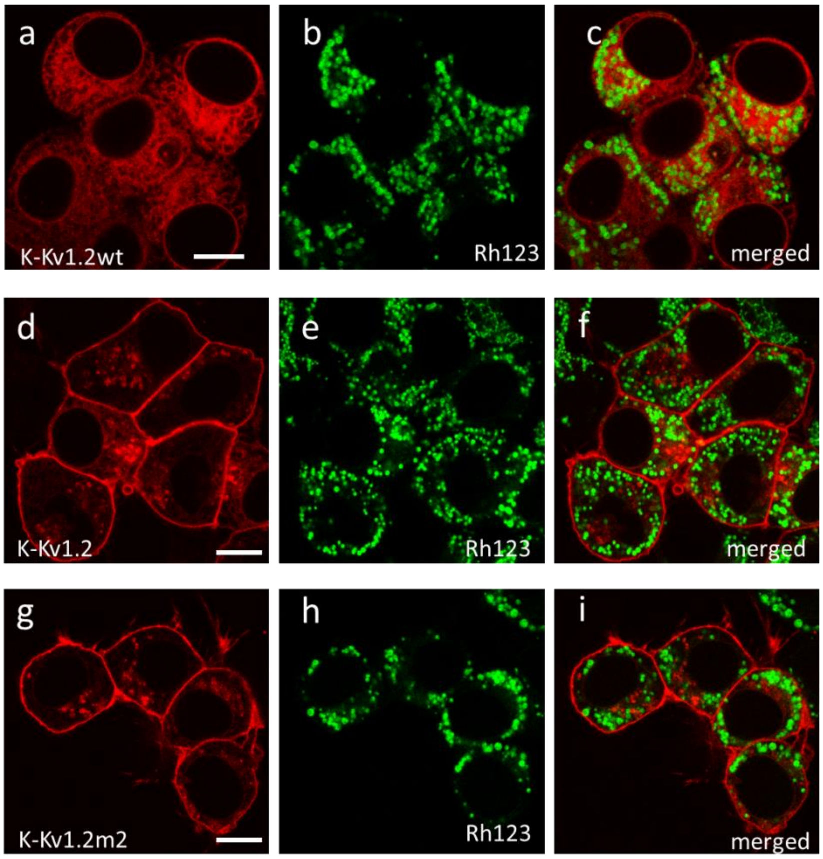

Figure S4. Analysis of localization of K-Kv1.2wt, K-Kv1.2 and K-Kv1.2m2 in mitochondria of Neuro 2a cells. (a,d,g) Confocal images of a distribution of K-Kv1.2wt (a), K-Kv1.2 (d) and K-Kv1.2m2 (g) in cells. (b,e,h) Confocal images of a distribution of the fluorescent marker of mitochondria (Rh123) in cells. (c,f,i) Merged images of Rh123 and Kv1.2 channels. In the case of the co-localization of Kv1.2 and Rh123 in mitochondria, an appearance of yellow color is expected. Bar is 20 µm.

Studies of affinity of HgTx-G to Kv1.1 and Kv1.3 channels

Affinity of HgTx-G to Kv1.1 and Kv1.3 channels was studied using previously engineered K-Kv1.1 and K-Kv1.3 channels (Kv1.1 and Kv1.3 channels fused with mKate2) expressed in the membrane of Neuro2a cells [1,2]. Experiments were performed similarly as described for the titration of K-Kv1.2 channels with HgTx-G, and the binding of HgTx-G to the channels at the cell membrane was analysed as described in the *Materials and Methods* section. For the complexes of HgTx-G with Kv1.1 and Kv1.3 channels dissociation constants are equal to 0.4±0.2 and 0.06±0.03 nM, respectively (Figure S5).

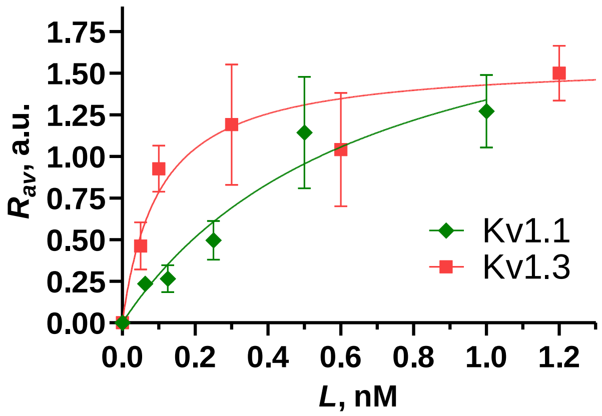

Figure S5. Concentration dependences of HgTx-G binding to recombinant Kv1.1 and Kv1.3 channels engineered and expressed in Neuro2a cells as described earlier [1,2]. The data are presented in terms of the dependence of the R_{av} parameter (see the *Materials and Methods* section) on the concentration L of HgTx-G added to cells. Data are averaged over three independent experiments and presented as mean±SEM.

Molecular modeling of the structure of the HgTx1, Ce1 and Ce4 peptides

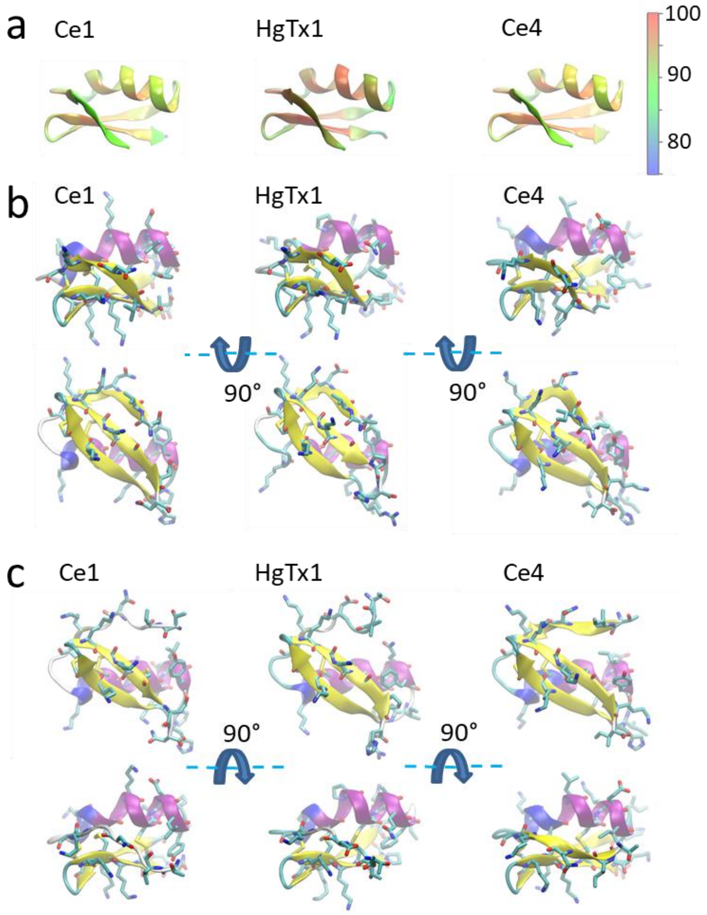

Figure S6. Structures of Ce1, Ce4 and HgTx1 predicted by AlfaFold2 (a) and further subjected to molecular dynamics relaxation (b, c). (a) Color scale corresponds to a model confidence score according to the Local Distance Difference Test (pLDDT).

Content of secondary structures in the studied peptides

Table S1. Content of secondary structures in the studied peptides (%) according to the CD analysis.

Peptide	α-helix	β-sheet	Turns	Random coil
Ce1	19	30	21	30
Ce4	17	33	20	30
HgTx1	19	33	22	26

Studies of affinity of Ce1 and Ce4 peptides to Kv1.1 and Kv1.3 channels

Affinity of MgTx to Kv1.3 channel and peptides Ce1 and Ce4 to Kv1.1 and Kv1.3 channels was studied using previously developed analytical systems based on fluorescent ligand A-HgTx and Kv1.1 (Kv1.3) channels expressed in the membrane of Neuro2a cells in the form of the conjugates with mKate2 fluorescent protein (K-Kv1.1 and K-Kv1.3) [1,2]. The approach based on the competitive displacement of A-HgTx (2 nM) from the complex with the channel by the increasing concentration of the studied peptide (Figure S6) and the previously approved formalism (described in the *Materials and Methods* section) were used to evaluate K_{ap} values for MgTx, Ce1 and Ce4 peptides. For the complexes of MgTx, Ce1 and Ce4 with Kv1.3 channel the K_{ap} values are 1.4±0.7, 13±8 and 30±10 nM, respectively. For the complex of Ce1 with Kv1.1 channel, K_{ap} is equal to 11±5 nM. Since Ce4 does not displace A-HgTx (2 nM) from the complex with Kv1.1 channel till the 1 μM concentration (Figure S6b), K_{ap} of the complex between Ce4 and Kv1.1 is estimated to be higher than 300 nM.

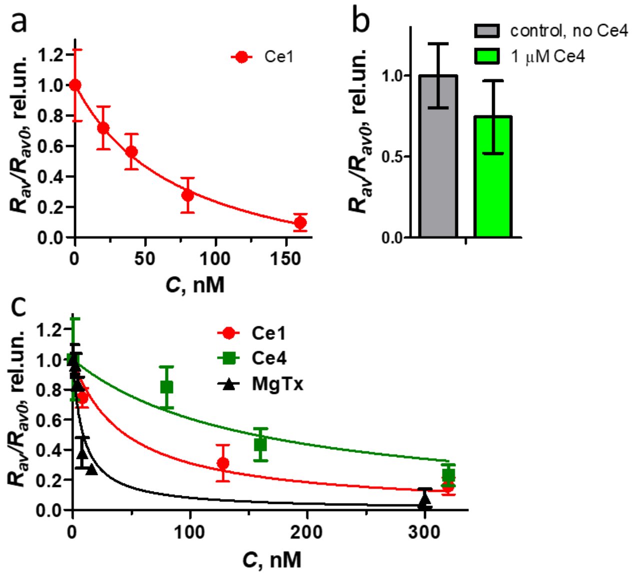

Figure S7. Competitive displacement of A-HgTx (2 nM) from the complexes with K-Kv1.1 (a, b) and K-Kv1.3 (c) by Ce1, Ce4 and MgTx peptides. (a,c) The dependences of the R_{av}/R_{av0} parameter (see the *Materials and Methods* section) on the concentration C of the added competitor are shown. Data are averaged over three independent experiments and presented as mean±SEM. (b) Histogram showing the effect of 1 µM Ce4 on the binding of A-HgTx (2 nM) to K-Kv1.1.

Molecular modeling of Kv1.2 channel and its complexes with peptide blockers

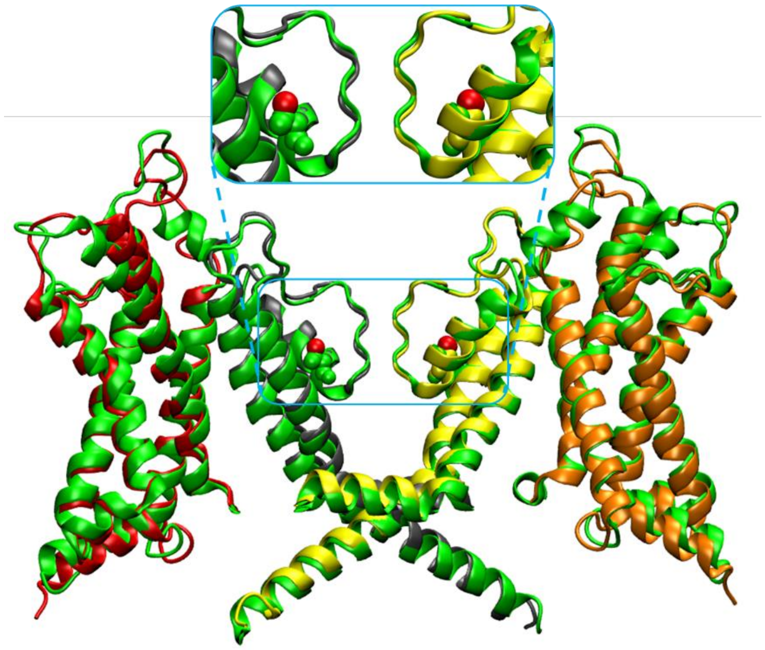

Figure S8. Side view of the merged models of the Kv1.2 channel (green color) and Kv1.2(S371T) channel (different subunits are shown in different colors). Residue 371 is shown in ball presentation. Zoom of the region of the selective filter and location of residue 371 is shown above the channel. For clarity, the back and front polypeptide chains are not shown. Lipid, water and ion molecules are not shown.

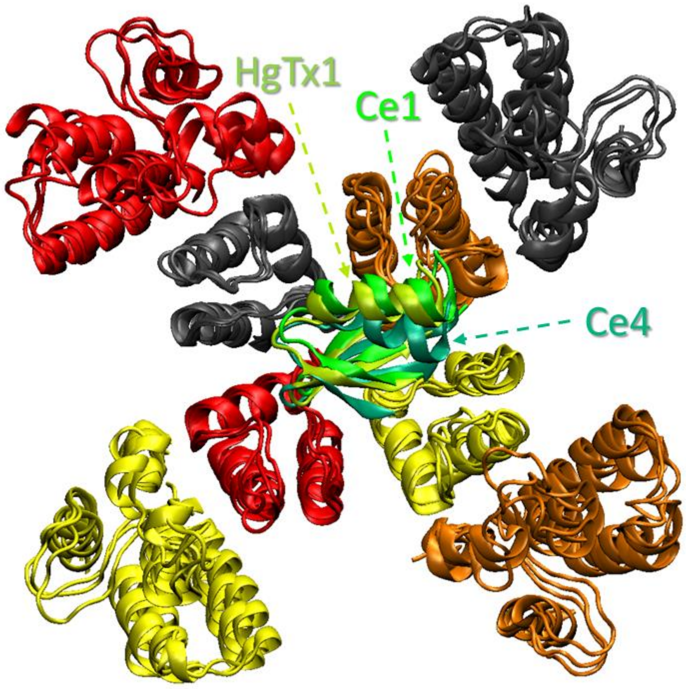

Figure S9. Merged models of the complexes between Kv1.2 channel and peptides Ce1, Ce4 and HgTx1 (view from the external surface of the membrane). The merging of the models was performed using amino acid residues 350-380 of the channel. The polypeptide chains of four α-subunits of the channel are shown in different colors: red, grey, brown and yellow. The polypeptide chains of Ce1, Ce4, and HgTx1 are represented in light green, green, and turquoise, respectively. Lipid, water and ion molecules are not shown.

References

1. Orlov, N.A.; Ignatova, A.A.; Kryukova, E. V.; Yakimov, S.A.; Kirpichnikov, M.P.; Nekrasova, O. V.; Feofanov, A. V. Combining mKate2-Kv1.3 Channel and Atto488-Hongotoxin for the Studies of Peptide Pore Blockers on Living Eukaryotic Cells. Toxins (Basel) 2022, 14, doi:10.3390/TOXINS14120858.
2. Orlov, N.A.; Kryukova, E. V.; Efremenko, A. V.; Yakimov, S.A.; Toporova, V.A.; Kirpichnikov, M.P.; Nekrasova, O. V.; Feofanov, A. V. Interactions of the Kv1.1 Channel with Peptide Pore Blockers: A Fluorescent Analysis on Mammalian Cells. Membranes (Basel) 2023, 13, doi:10.3390/MEMBRANES13070645.
